# A Polar Flagellar Transcriptional Program Mediated by Diverse Two-Component Signal Transduction Systems and Basal Flagellar Proteins Is Broadly Conserved in Polar Flagellates

**DOI:** 10.1128/mBio.03107-19

**Published:** 2020-03-03

**Authors:** Peter M. Burnham, William P. Kolar, David R. Hendrixson

**Affiliations:** a Department of Microbiology, University of Texas Southwestern Medical Center, Dallas, Texas, USA; Yale University School of Medicine; University of Georgia

**Keywords:** *Campylobacter jejuni*, FlhF, FlhG, MS ring, *Pseudomonas aeruginosa*, T3SS, *Vibrio cholerae*, flagellar biogenesis, motility, polar flagellation, two-component signal transduction

## Abstract

Bacterial flagella are rotating nanomachines required for motility. Flagellar gene expression and protein secretion are coordinated for efficient flagellar biogenesis. Polar flagellates, unlike peritrichous bacteria, commonly order flagellar rod and hook gene transcription as a separate step after production of the MS ring, C ring, and flagellar type III secretion system (fT3SS) core proteins that form a competent fT3SS. Conserved regulatory mechanisms in diverse polar flagellates to create this polar flagellar transcriptional program have not been thoroughly assimilated. Using *in silico* and genetic analyses and our previous findings in Campylobacter jejuni as a foundation, we observed a large subset of Gram-negative bacteria with the FlhF/FlhG regulatory system for polar flagellation to possess flagellum-associated two-component signal transduction systems (TCSs). We present data supporting a general theme in polar flagellates whereby MS ring, rotor, and fT3SS proteins contribute to a regulatory checkpoint during polar flagellar biogenesis. We demonstrate that Vibrio cholerae and Pseudomonas aeruginosa require the formation of this regulatory checkpoint for the TCSs to directly activate subsequent rod and hook gene transcription, which are hallmarks of the polar flagellar transcriptional program. By reprogramming transcription in V. cholerae to more closely follow the peritrichous flagellar transcriptional program, we discovered a link between the polar flagellar transcription program and the activity of FlhF/FlhG flagellar biogenesis regulators in which the transcriptional program allows polar flagellates to continue to produce flagella for motility when FlhF or FlhG activity may be altered. Our findings integrate flagellar transcriptional and biogenesis regulatory processes involved in polar flagellation in many species.

## Abstract (precis)

Relative to peritrichous bacteria, polar flagellates possess regulatory systems that order flagellar gene transcription differently and produce flagella in specific numbers only at poles. How transcriptional and flagellar biogenesis regulatory systems are interlinked to promote the correct synthesis of polar flagella in diverse species has largely been unexplored. We found evidence for many Gram-negative polar flagellates encoding two-component signal transduction systems with activity linked to the formation of flagellar type III secretion systems to enable production of flagellar rod and hook proteins at a discrete, subsequent stage during flagellar assembly. This polar flagellar transcriptional program assists, in some manner, the FlhF/FlhG flagellar biogenesis regulatory system, which forms specific flagellation patterns in polar flagellates in maintaining flagellation and motility when activity of FlhF or FlhG might be altered. Our work provides insight into the multiple regulatory processes required for polar flagellation.

## INTRODUCTION

Many bacteria synthesize flagella for swimming motility. Each species produces a specific flagellation pattern defined by the spatial arrangement and number of flagella presented on the cell surface. Peritrichous flagellates construct many flagella across the surface, whereas polar flagellates generate flagella only at polar regions. Polar flagellates are further categorized by the number of flagella per cell: monotrichous (one flagellum at one pole), amphitrichous (one flagellum at each pole), and lophotrichous (a tuft of a few flagella at one pole).

Flagellar placement and number in many polar flagellates are controlled by the FlhF GTPase and FlhG/FleN ATPase (1, 2). FlhF is hypothesized to function in some polar flagellates in a GTP-bound “on” state to perform an unknown essential step for flagellar biogenesis at a pole. Transitioning to a GDP-bound “off” state upon GTP hydrolysis may limit the production of additional polar flagella. In some polar flagellates, FlhG stimulates FlhF GTPase activity *in vitro*, which has been proposed to influence flagellum numbers by controlling *in vivo* FlhF-dependent polar flagellation activities (3–6). However, FlhG orthologs in other species control polar flagellar number by repressing the activity or expression of a specific master transcriptional regulator so that an ideal level of flagellar genes sufficient to produce the correct number of flagella are expressed (7–9). Many molecular details for how FlhF and FlhG control polar flagellation remain elusive. It is anticipated that FlhF and FlhG activities vary among species, resulting in different flagellation patterns in polar flagellates.

Despite different flagellation patterns, many peritrichous and polar flagellates possess some conserved strategies to coordinate flagellar gene transcription with stages of flagellar assembly (10–13). These strategies allow for tight regulation of ordered flagellar protein secretion that is conducive to flagellar motor biogenesis. Stages of flagellar assembly can be marked by distinct cues or regulatory checkpoints that are detected by different mechanisms to stimulate gene transcription and protein production to complete the next stage of assembly. Flagellar biogenesis begins by activating the transcription of genes encoding components essential for the initial steps in assembly, which include the flagellar type III secretion system (fT3SS), MS ring, and C ring rotor and switch proteins (14–20). MS and C ring formation around the fT3SS core completes biogenesis of a competent fT3SS for export and assembly of rod and hook components (21–25). Up to this point, the alternative σ factor σ^28^, which is required for transcription of flagellins and other motility genes, is inhibited by the anti-σ factor FlgM (11, 13, 26–29). Hook biogenesis completes a regulatory checkpoint that facilitates an fT3SS substrate specificity switch to secrete FlgM out of the cell via the fT3SS (11, 13, 30). Derepression of σ^28^ allows for transcription of genes that complete flagellar filament polymerization and motor assembly.

**FIG 1:**
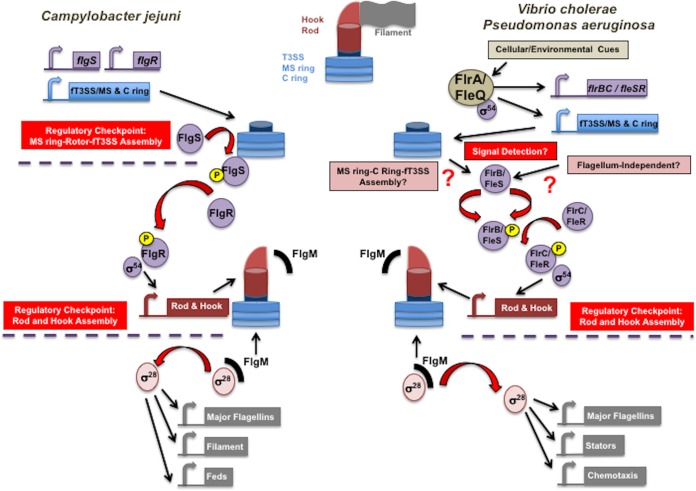
Defined and unknown regulatory steps of the polar flagellar transcriptional program of C. jejuni, V. cholerae, and P. aeruginosa. Simplified models of transcriptional regulation of different classes of flagellar genes in the Gram-negative polar flagellates C. jejuni, V. cholerae, and P. aeruginosa. V. cholerae and P. aeruginosa (right) initiate flagellar gene transcription via master transcriptional regulators (FlrA and FleQ, respectively) to transcribe an initial class of flagellar genes; C. jejuni lacks a master transcriptional regulator and expression of this class of flagellar genes appears to be constitutive (left). These initial flagellar genes encode the fT3SS, MS ring, C ring, and a flagellum-associated TCS (FlgSR in C. jejuni, FlrBC in V. cholerae, and FleSR in P. aeruginosa). Expression of the C. jejuni flagellar rod, ring, and hook genes is dependent upon σ^54^, the FlgSR TCS, and the fT3SS, FliF MS ring, and FliG C ring rotor proteins (31–33). The formation of the MS ring-rotor-fT3SS complex is an early regulatory checkpoint sensed by the FlgS sensor kinase to initiate phosphotransfer to the FlgR response regulator, which allows FlgR to function with σ^54^ for rod and hook gene expression. (Right) Exploration of factors that may form a similar regulatory checkpoint during flagellar assembly that are required for the activity of the V. cholerae FlrBC TCS and the P. aeruginosa FleSR TCS addressed in this work. A regulatory checkpoint during late flagellar assembly occurs upon formation of the flagellar rod and hook in all of these organisms and many other peritrichous and polar flagellates. Completion of rod and hook assembly promotes a substrate specificity switch that facilitates secretion of the anti-σ factor FlgM from the cytoplasm and derepression of σ^28^ for transcription of flagellins and other proteins that complete flagellar biogenesis for motor function. C. jejuni also contains *fed* genes that are dependent on σ^28^ for expression and are not involved in motility but are required for the colonization of avian species and some virulence processes (92, 93).

We previously explored how the amphitrichous polar flagellate Campylobacter jejuni coordinates the transcription of flagellar genes with flagellar assembly (Fig. 1) (31–33). We discovered that fT3SS core proteins (FlhA, FlhB, FliP, FliQ, and FliR), the FliF MS ring, and FliG rotor of the C ring assemble into the MS ring-rotor-fT3SS complex to form a regulatory checkpoint monitored by C. jejuni. We found the flagellum-associated FlgSR two-component signal transduction system (TCS) detects MS ring-rotor-fT3SS formation to directly activate σ^54^-dependent flagellar rod and hook gene expression (Fig. 1) (31–33). As a means of signal detection, we observed that the cytoplasmic FlgS sensor kinase of the FlgSR TCS physically interacted with FliF and FliG only after these proteins multimerized into the MS ring and rotor around the fT3SS core (Fig. 1) (32). In mutants defective for MS ring-rotor-fT3SS complex assembly, FlgS did not interact with FliF and FliG. Currently, it is not known whether FlgS interacts with surfaces of adjacent FliF subunits, FliG subunits, or FliF-FliG complexes of the MS ring-rotor structure surrounding the fT3SS core. Detection of this regulatory checkpoint by an orthologous FlgSR TCS also may occur in Helicobacter pylori, a lophotrichous epsilonproteobacterium closely related to C. jejuni, for transcription of σ^54^-dependent rod and hook genes (34–37). Genetic analyses indicate that H. pylori FlgSR TCS activity for rod and hook gene transcription is also dependent on fT3SS, MS ring, and C ring proteins, suggesting that this TCS in H. pylori also senses the formation of a competent fT3SS (38–43).

Vibrio cholerae and Pseudomonas aeruginosa are monotrichous polar flagellates that also produce a flagellum-associated TCS. The V. cholerae FlrBC and P. aeruginosa FleSR TCSs, like the C. jejuni FlgSR TCS, are directly required with σ^54^ for flagellar rod and hook gene expression (17–19, 44, 45). As with C. jejuni FlgSR, signal transduction through V. cholerae and P. aeruginosa TCSs results in phosphorylation of the cognate FlrC and FleR response regulators to promote their binding to rod and hook gene promoters and directly assist σ^54^ in activating transcription of rod and hook genes (44–47). Although transcription of the V. cholerae and P. aeruginosa FlrBC and FleSR TCSs occurs simultaneously with fT3SS, MS ring, and C ring genes by a master transcriptional regulator (18, 19), it is not known whether these TCSs sense cues associated with flagellar assembly or flagellum-independent cellular cues to initiate and coordinate flagellar rod and hook gene expression with a stage of flagellar assembly (Fig. 1). Transcription of rod and hook genes after expression of MS ring, C ring, and fT3SS components appears to comprise a polar flagellar transcriptional program not observed in peritrichous flagellates.

Noticeably, many peritrichous flagellates appear to ignore the formation of the MS ring-rotor-fT3SS complex as a regulatory checkpoint that we discovered in C. jejuni and is likely present in H. pylori. The model peritrichous bacteria Escherichia coli and *Salmonella* lack a flagellum-associated TCS and do not employ σ^54^ for flagellar gene transcription. These bacteria also do not require the MS ring, C ring, and fT3SS proteins for rod and hook gene expression (10, 15, 48). Instead, these bacteria express fT3SS, MS ring, and C ring proteins simultaneously with flagellar rod and hook genes, which we designate, for the purposes of this report, the peritrichous flagellar transcriptional program. Hence, a competent fT3SS is not required for expression of rod and hook genes in peritrichous flagellates, in contrast to C. jejuni and H. pylori. These differences raise intriguing questions for flagellar biogenesis in polar flagellates. (i) How common is it for polar flagellates to possess flagellum-associated TCSs? (ii) Do polar flagellates with flagellum-associated TCSs broadly require MS ring, C ring, and fT3SS proteins for activity to result in rod and hook gene expression? (iii) Do polar flagellates employ the respective sensor kinases to detect a regulatory checkpoint formed by the MS ring, C ring, and/or fT3SS? (iv) Do some TCSs detect flagellum-independent cues to activate rod and hook gene expression? (v) Is the polar flagellar transcriptional program that separates production of fT3SS, MS ring, and C ring proteins from rod and hook proteins required for or beneficial to a specific process in polar flagellates to build flagella?

We evaluated the conservation of regulatory systems that order gene expression for a polar flagellar transcriptional program in diverse polar flagellates. Our results, combined with previous findings in alphaproteobacteria, indicate a broad, common theme in polar flagellates, whereby different mechanisms are employed to coordinate rod and hook protein production with a stage of flagellar assembly involving the formation of a competent fT3SS. We found that a large subset of polar flagellates with FlhF/FlhG flagellar biogenesis regulatory systems encode orthologous flagellum-associated TCSs. Additional evidence combined with our previous findings suggests that these TCSs have a conserved function in detecting a similar regulatory checkpoint centered around MS ring-rotor-fT3SS complex formation. Our findings suggest that the polar flagellar transcriptional program, rather than a peritrichous one, allows polar flagellates to sustain flagellation and motility if FlhF and/or FlhG activity is altered and provide speculation into the evolution of polar flagellates. Our work provides insight into connections between flagellar transcriptional and biogenesis regulatory systems involved in polar flagellation.

## RESULTS

### A large subset of gram-negative polar flagellates possesses flagellum-associated TCSs.

We followed a bioinformatic strategy to evaluate the prevalence of flagellum-associated TCS in a broad range of Gram-negative polar flagellates. We limited our analysis to Gram-negative bacteria to enable comparisons with previous studies in bacteria that employ flagellum-associated TCSs for direct activation of σ^54^-dependent flagellar rod and hook gene expression, such as C. jejuni, H. pylori, V. cholerae, and P. aeruginosa. For this approach, we performed reciprocal best hit sequence alignments with a set of 117 reference bacterial genomes available at NCBI Assembly to identify Gram-negative flagellates. Genomes included in this reference set were curated by NCBI to represent high-quality, community-standard bacterial genomes or genomes of medically relevant bacteria. Although not every sequenced bacterial genome is included in this reference set, it is sufficient to survey and acquire information regarding features present among phylogenetically diverse bacteria.

**FIG 2:**
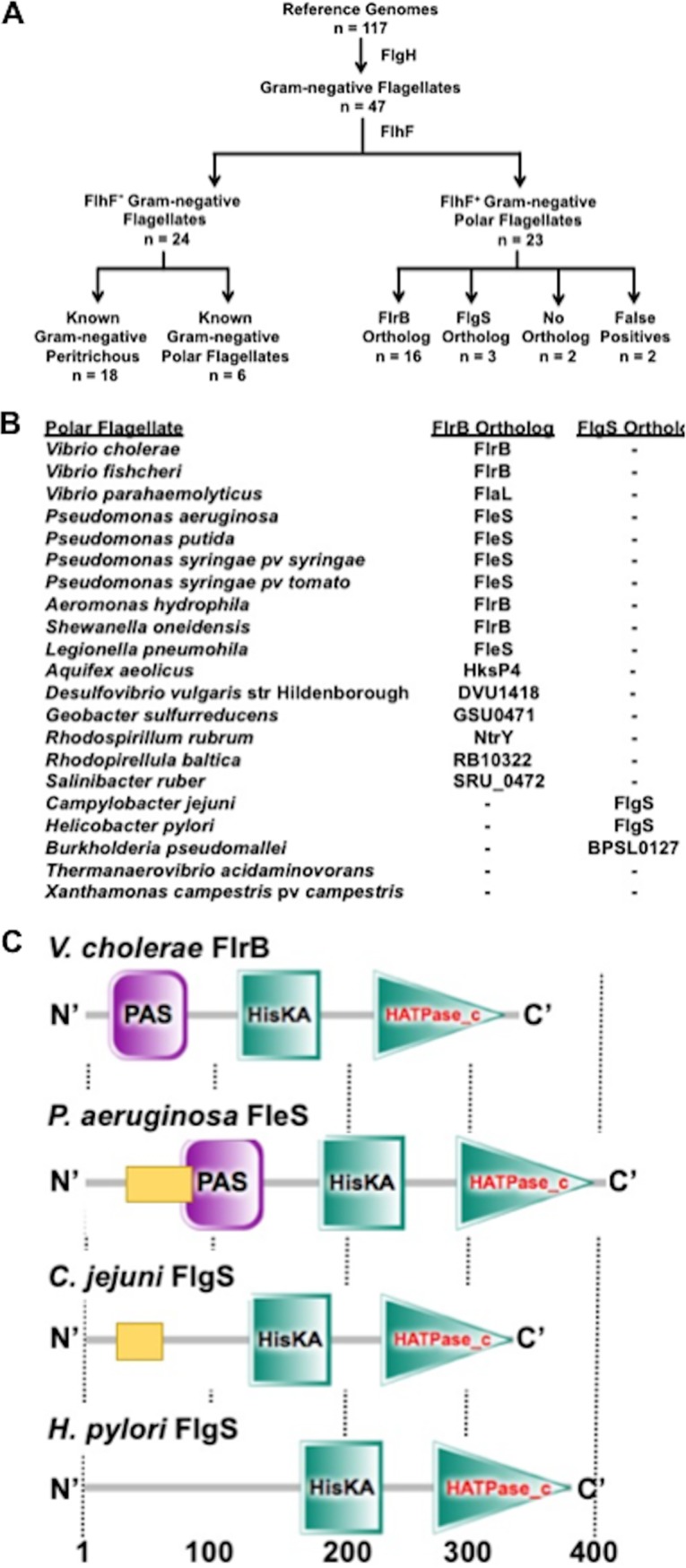
Bioinformatic analysis of predicted Gram-negative polar flagellates and flagellum-associated TCSs. (A) Flowchart of the strategy and outcomes of tBLASTn reciprocal best hit sequence alignments of predicted Gram-negative polar flagellates from a reference set of bacterial genomes and those with V. cholerae FlrB or C. jejuni FlgS orthologs. (B) Sensor kinases of putative flagellum-associated TCSs of predicted Gram-negative polar flagellates. For each polar flagellate, the annotated or predicted kinase is indicated as an FlrB or FlgS ortholog depending on the resultant score. (C) Domain analysis of V. cholerae FlrB, P. aeruginosa FleS, C. jejuni FlgS, and H. pylori FlgS as predicted by SMART. The conserved HisKA and HATPase_C domains of bacterial sensor kinases for histidine autophosphorylation, dimerization, and ATPase activity are shown in turquoise squares and triangles, respectively. Putative predicted PAS domains are shown as purple squares, and predicted coiled-coil domains are shown as gold rectangles. Numbers below indicate approximate positions of amino acids.

We first used E. coli FlgH as a marker for Gram-negative bacteria producing flagella (Fig. 2A). FlgH forms the L-ring required for external flagella to penetrate the outer membrane barrier in Gram-negative flagellates (49–51). Since FlhF is involved in polar flagellation, we then used V. cholerae FlhF as a marker to predict polar flagellates within the Gram-negative flagellates (Fig. 2A) (1, 2, 7). Our results initially identified 23 of 47 putative Gram-negative flagellates within the reference set as polarly flagellated species (Fig. 2B), with two clear false positives: Bordetella bronchiseptica, which encodes a predicted FlhF (BN112_0372) but is a known peritrichous organism (52), and Burkholderia mallei, which harbors mutations in multiple flagellar genes and is likely undergoing reductive genome evolution (53). Twenty of 21 predicted Gram-negative polar flagellates encoded a predicted FlhG ortholog immediately downstream of *flhF*, indicating that an FlhF/FlhG flagellar biogenesis regulatory system was intact; only Rhodospirillum rubrum lacked an *flhG* ortholog organized with *flhF*. Of the 24 predicted Gram-negative flagellates lacking FlhF, 18 are known peritrichous organisms. The remaining six are actually polar flagellates with five species from alphaproteobacteria, best represented by Caulobacter crescentus, which employs factors other than FlhF for polar flagellation. Thus, FlgH and FlhF were relatively robust predictors of Gram-negative polar flagellates within the reference set except for alphaproteobacterial species.

From the 21 remaining predicted Gram-negative polar flagellates with FlhF orthologs, we performed reciprocal best hit sequence alignments using the V. cholerae FlrB sensor kinase of the flagellum-associated FlrBC TCS to identify 16 species with flagellum-associated TCSs (Fig. 2A and B). Although this approach identified putative flagellum-associated TCS sensor kinases in many polar flagellates (including FleS of the FleSR TCS of different *Pseudomonas* species), C. jejuni FlgS was not identified. We repeated our reciprocal best hit sequence alignment analysis using C. jejuni FlgS. C. jejuni FlgS did not identify FlrB or FleS as orthologs but did identify flagellum-associated sensor kinases in two of the four predicted polar flagellates without FlrB orthologs, H. pylori FlgS and Burkholderia pseudomallei BPSL0127 (Fig. 2A and B). These observations suggest that sensor kinases of flagellum-associated TCSs in polar flagellates are divided into distinct FlrB-like or FlgS-like groups. Overall, our bioinformatic analysis indicated that 19 of 21 predicted Gram-negative polar flagellates from the reference set encode both an FlhF/FlhG regulatory system and a putative flagellum-associated TCS. Thus, our results suggest a high degree of cooccurrence between the two regulatory systems exist in bacterial species that produce polar flagella. The only two Gram-negative FlhF/FlhG-positive species in the reference set that did not encode a flagellum-associated TCS were the known polar flagellate *Xanthamonas campestris* pv. *campestris*, previously noted to lack a respective TCS, and Thermanaerovibrio acidaminovorans, whose prediction as a polar flagellate may be dubious, as it produces lateral flagella on its concave surface (54, 55) (Fig. 2B).

To ensure that our FlrB homologs are flagellum-associated sensor kinases rather than conserved unrelated sensor kinases involved in other processes, we examined the correlation of V. cholerae sensor kinases with the presence of FlhF across genomes in the reference set (see Fig. S1 in the supplemental material). If non-flagellum-associated sensor kinases are within our predicted FlrB orthologs, we would expect a weak to negative correlation of these kinases with FlhF that is indistinguishable from the correlation of a random V. cholerae sensor kinase to FlhF. In contrast, if FlrB orthologs are flagellum-associated TCS kinases, we would expect V. cholerae FlrB to be one of the kinases most highly correlated with FlhF. We found that predicted FlrB orthologs had a stronger positive correlation to V. cholerae FlhF than all but two of 51 V. cholerae sensor kinases (Fig. S1). One of these kinases is V. cholerae CheA, which is the major sensor kinase in the chemotaxis system that influences flagellar rotation and motility in many bacterial species (56, 57). The other V. cholerae kinase is VCA0851, an uncharacterized kinase. These results support our flagellum-associated TCS predictions and further emphasize the correlation between the FlhF/FlhG flagellar biogenesis system and the flagellum-associated TCSs.

10.1128/mBio.03107-19.4 FIG S1 Correlation of V. cholerae sensor kinase orthologs in predicted polar flagellates to V. cholerae FlhF orthologs. The relative correlation of each set of kinase orthologs for the 51 V. cholerae sensor kinases taken from each of the reference genomes to FlhF is shown as a Pearson correlation coefficient. Values closer to 1 indicate that the sensor kinase orthologs are more positively correlated with FlhF orthologs. Values greater than 0.4 are considered significant positive correlations. The score for FlrB orthologs is indicated in red. Download FIG S1, TIF file, 1.1 MB. Copyright © 2020 Burnham et al. 2020 Burnham et al. This content is distributed under the terms of the Creative Commons Attribution 4.0 International license.

Our bioinformatic analysis suggested that the sensor kinases in flagellum-associated TCS systems of Gram-negative polar flagellates belong to two or more unrelated groups and possess different features, since C. jejuni FlgS and V. cholerae FlrB did not identify each other as orthologs. Many of these sensor kinases are predicted to be cytoplasmic kinases lacking transmembrane domains. Representative kinases such as C. jejuni FlgS, H. pylori FlgS, V. cholerae FlrB, and P. aeruginosa FleS contain similar HisKA and HATPase_c domains that function in histidine autophosphorylation, dimerization, and ATPase activity but have divergent N-terminal sensor domains (Fig. 2C) (58, 59). Both V. cholerae FlrB and P. aeruginosa FleS contain a predicted PAS domain that is a common sensing domain in sensor kinases; FleS also has a predicted coiled-coil domain for potential protein interactions (Fig. 2C) (59). However, the only predicted domain within the C. jejuni FlgS sensor region is a coiled-coil domain, and no predicted structural domain was identified in H. pylori FlgS (Fig. 2C). These observations suggest that flagellum-associated sensor kinases within FlhF/FlhG-containing polar flagellates detect different cellular signals or detect similar signals by different mechanisms to initiate signal transduction for flagellar gene expression and polar flagellar biogenesis.

### The V. cholerae FlrBC TCS requires the fT3SS, MS ring, and rotor for activity.

As described above, we previously discovered that C. jejuni FlgS detects MS ring-rotor-fT3SS assembly by a direct interaction as a regulatory checkpoint to initiate signal transduction for FlgSR- and σ^54^-dependent transcription of rod and hook genes for the polar flagellar transcriptional program (Fig. 1) (32). The V. cholerae and P. aeruginosa flagellum-associated FlrBC/FleSR TCSs are expressed simultaneously with the fT3SS, MS ring, and C ring genes. However, investigations to identify what signals are detected by these TCSs to initiate signal transduction for TCS- and σ^54^-dependent flagellar rod and hook gene expression that occurs in the subsequent tier of the polar flagellar transcriptional program are lacking (Fig. 1) (18, 19). Considering that the sensor domains of V. cholerae FlrB and P. aeruginosa FleS differ from that of C. jejuni FlgS, we hypothesized that the FlrB and FleS kinases detect a similar regulatory checkpoint formed by fT3SS, MS ring, and/or C ring components but by different means, or they detect a signal independent of flagellar biogenesis (Fig. 1). Thus, we first investigated whether disruption of the fT3SS, MS ring, and/or C ring impacted the activity of the V. cholerae FlrBC TCS for σ^54^-dependent rod and hook gene expression.

Transcriptional fusions of the promoters of four V. cholerae FlrBC- and σ^54^-dependent operons, *flgBCDE*, *flgFGHIJ*, *flgKL*, and *flaA*, to a promoterless *lacZ* gene were created in WT V. cholerae and isogenic flagellar mutants. The former three operons encode rod and hook genes, whereas *flaA* encodes the major flagellin. V. cholerae is different from many other flagellates in that the major flagellin is not expressed from a σ^28^-dependent promoter; instead, minor flagellins are expressed from a σ^28^-dependent promoter in this bacterium (60). Of note, the *flgFGHIJ* rod operon was previously proposed to be cotranscribed with the *flgBCDE* rod and hook operon from the *flgB* promoter (18). However, we identified a putative σ^54^ binding site between *flgE* and *flgF* and constructed an *flgFp*-*lacZ* transcriptional fusion for analysis. A *cheVp*-*lacZ* transcriptional fusion served as a control, as *cheV* expression is independent of FlrBC TCS and σ^54^ (18).

**FIG 3:**
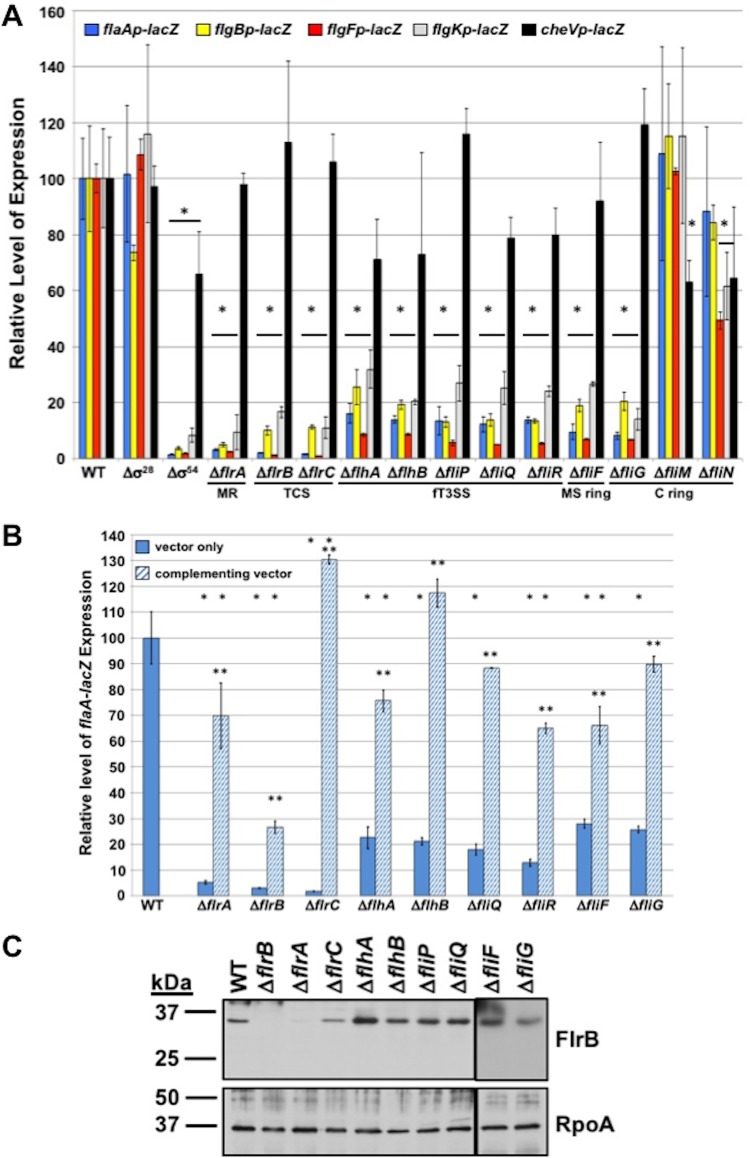
Analysis of TCS- and σ^54^-dependent flagellar gene expression in flagellar mutants of V. cholerae. (A) Expression of flagellar rod and hook operons in WT V. cholerae C6706 and isogenic flagellar mutants. *flgBp*-, *flgFp*-, *flgKp*-, *flaAp*-, and *cheVp*-*lacZ* transcriptional reporters were maintained on plasmids in V. cholerae strains. The level of expression of each transcriptional reporter in each mutant is relative to the level of expression in WT V. cholerae, which was set to 100 U. MR, master regulator; TCS, two-component signal transduction system. (B) Expression of *flaAp*-*lacZ* transcriptional fusion in WT V. cholerae and an isogenic mutant containing vector alone (solid blue bars) or vectors to express genes from a constitutive promoter for complementation (hatched blue bars). The level of expression of transcriptional reporters in each mutant is relative to the level of expression in WT V. cholerae with vector alone, which was set to 100 U. For panels A and B, results are from a representative assay with each sample analyzed in triplicate. Error bars indicate standard deviations of the average level of expression from three samples. An asterisk indicates significant difference in expression from the WT (A) or WT containing vector alone (B) (*P* < 0.05). Two asterisks indicate significant increase in expression from the respective mutant containing vector only (*P* < 0.05). (C) Immunoblot analysis of the FlrB sensor kinase levels in whole-cell lysates of WT V. cholerae and isogenic mutants. Specific antiserum to FlrB was used to detect the protein. Detection of RpoA served as a control to ensure equal loading of proteins across strains.

We verified previous findings that V. cholerae Δ*flrA*, Δσ^54^ Δ*flrB*, and Δ*flrC* (but not Δσ^28^) mutants were defective for *flaAp*-, *flgBp*-, and *flgKp*-*lacZ* expression (Fig. 3A) (18, 45); expression of these reporters was reduced 6- to >50-fold in these mutants relative to that of WT V. cholerae. The defect in Δ*flrA* is due to FlrA functioning as a master transcriptional regulator required for *flrBC* expression (Fig. 1) (17, 18). We also confirmed that an FlrBC- and σ^54^-dependent promoter upstream of *flgF* drives expression of *flgFGHIJ* independently of *flgBp*; *flgFp*-*lacZ* expression was decreased 50- to 100-fold in FlrBC TCS or σ^54^ mutants (Fig. 3A).

We next discovered that V. cholerae mutants lacking fT3SS core proteins (including FlhA, FlhB, FliP, FliQ, and FliR) and the FliF MS ring protein were defective for FlrBC activity and rod and hook transcription, as expression of the respective transcriptional reporters was reduced 3- to 20-fold (Fig. 3A). The C ring FliG rotor protein was also required for FlrBC- and σ^54^-dependent flagellar gene expression, but the C ring FliM and FliN switch proteins were not (Fig. 3A). These results are similar to our findings for requirements for C. jejuni FlgSR TCS directly activating σ^54^-dependent flagellar rod and hook gene expression (Fig. S2) (31–33). Either no reductions or only modest reductions in *cheVp*-*lacZ* expression were observed in these flagellar mutants. *In trans* complementation of the V. cholerae fT3SS, *fliF*, and *fliG* mutants with the respective gene restored expression of *flaAp*-*lacZ* to a significantly higher level than that of each mutant with vector alone (and to at least 60% of the level observed in the WT; Fig. 3B). We were unable to construct a complementing vector for the *fliP* mutant due to toxicity during attempted construction. *flaAp*-*lacZ* expression was partially or fully restored in *flrA*, *flrB*, and *flrC* mutants with complementation relative to the mutants with vector alone. Additionally, we verified that FlrB production was unaffected in mutants lacking the fT3SS, FliF, or FliG, which eliminated the possibility that the FlrBC TCS was unstable or not expressed in these mutants as an explanation of their reduction in rod and hook gene expression (Fig. 3C). The finding that a lack of individual MS ring, rotor, and fT3SS proteins abolishes FlrBC-dependent gene expression is consistent with our hypothesis that V. cholerae flagellar components form a regulatory checkpoint, possibly involving functional fT3SS assembly, required for FlrBC to activate rod and hook gene expression, as we previously demonstrated in C. jejuni (31–33).

10.1128/mBio.03107-19.5 FIG S2 C. jejuni FlgSR TCS activity for σ^54^-dependent flagellar gene expression in WT and flagellar mutants. Expression of the Δ*flaB*::*astA* transcriptional reporter in WT C. jejuni strain 81-176 and isogenic flagellar mutants is shown and comparable to our previously published analysis (32). The level of *flaB*::*astA* expression in each mutant is relative to the level of expression in WT C. jejuni, which was set to 100 U. Results from a representative assay with each sample analyzed in triplicate are shown. Error bars indicate standard deviations of the average levels of expression from three samples. An asterisk indicates the mutant had significantly increased or decreased reporter expression relative to that of the WT strain (*P* < 0.05). Download FIG S2, TIF file, 1.1 MB. Copyright © 2020 Burnham et al. 2020 Burnham et al. This content is distributed under the terms of the Creative Commons Attribution 4.0 International license.

### The P. aeruginosa FleSR TCS requires the fT3SS, MS ring, and rotor for activity.

**FIG 4:**
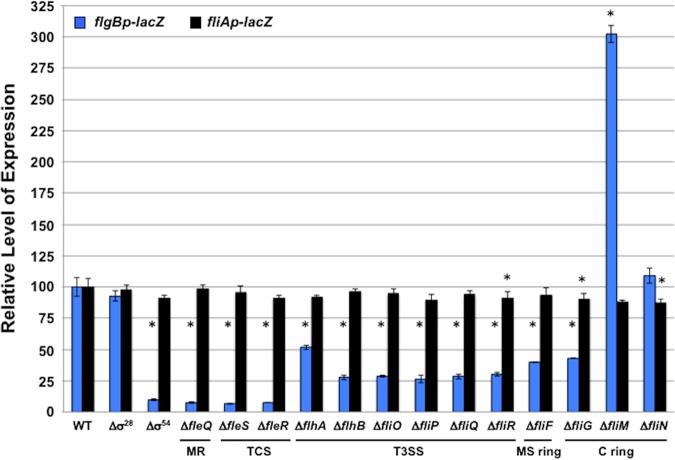
Analysis of TCS- and σ^54^-dependent flagellar gene expression in flagellar mutants of P. aeruginosa. Expression of the flagellar rod and hook operon in WT P. aeruginosa PA14 and isogenic flagellar mutants is shown. *flgBp*-*lacZ* and fliAp-*lacZ* transcriptional reporters were integrated at the *att* site on the chromosome of P. aeruginosa strains. The level of expression of transcriptional reporters in each mutant is relative to the level of expression in WT P. aeruginosa, which was set to 100 U. Results are from a representative assay, with each sample analyzed in triplicate. Error bars indicate standard deviations of the average level of expression from three samples. An asterisk indicates the mutant had a significantly increased or decreased reporter expression relative to that of the WT strain (*P* < 0.05). MR, master regulator; TCS, two-component signal transduction system.

We next investigated whether P. aeruginosa FleSR-dependent expression of flagellar rod and hook genes was influenced by fT3SS, MS ring, and C ring protein production and possibly assembly into an MS ring-rotor-fT3SS complex. We generated complete or partial in-frame deletion mutants of flagellar genes in P. aeruginosa PA14 and then integrated transcriptional fusions of the promoters of the *flgB* rod and hook operon and *fliA* (encoding σ^28^) to *lacZ* in the *att* site on the chromosome. We verified that transcription of the P. aeruginosa *flgB* rod and hook operon is dependent on σ^54^ and the FleSR TCS, in addition to the FleQ master transcriptional regulator that is required for *fleSR* transcription (Fig. 1 and 4) (19, 20). The *fliAp*-*lacZ* reporter served as a control, as *fliA* expression is independent of σ^54^ or the FleSR TCS (19). Deletion of σ^28^ did not affect expression of either reporter. We discovered that FleSR- and σ^54^-dependent expression of *flgBp*-*lacZ* in P. aeruginosa required the fT3SS, FliF MS ring, and the FliG C ring proteins, but not FliM or FliN, for full expression, similar to our analysis of the V. cholerae FlrBC and C. jejuni FlgSR TCSs (Fig. 3A and 4 and Fig. S2) (31–33). Expression of *fliAp*-*lacZ* was unaffected in these mutants. Thus, our data continue to support that many polar flagellates with the FlhF/FlhG flagellar biogenesis regulatory system contain flagellum-associated TCSs that require flagellar-dependent cues to stimulate expression of σ^54^-dependent flagellar rod and hook genes as a discrete step for the polar flagellar transcriptional program.

### Requirements of FliF and FliG for flagellum-associated TCS activity.

Having established that deletion of individual MS ring, rotor, and fT3SS proteins impeded FlrBC/FleSR TCS activity, we assessed whether these proteins alone or as a part of the MS ring-rotor-fT3SS complex were required for the activity of V. cholerae and P. aeruginosa TCSs to stimulate flagellar rod and hook gene transcription. Because FlrB and FleS are predicted to be cytoplasmic kinases, we hypothesized that they detect signals within the cytoplasm, similar to C. jejuni FlgS sensing formation of the MS ring and rotor by FliF and FliG via a direct interaction with the cytoplasmic domains of these structures (32). FliF is predicted to contain two transmembrane domains with a large central periplasmic domain and smaller N- and C-terminal cytoplasmic domains. A conserved periplasmic ASVXV motif in FliF is required for flagellation in *Salmonella* (61). This motif has been hypothesized to promote recruitment of FliF to the fT3SS core via interactions with FlhA and/or FliF multimerization into the MS ring around the fT3SS core. Alteration of the C. jejuni FliF ASVXV motif eliminated FlgS interactions with FliF and FliG and abolished FlgSR TCS signal transduction for rod and hook gene expression (32), supporting the hypothesis that FliF multimerization into the MS ring (and simultaneous rotor formation by FliG) around the complete fT3SS core is required to form a signal directly detected by FlgS.

**FIG 5:**
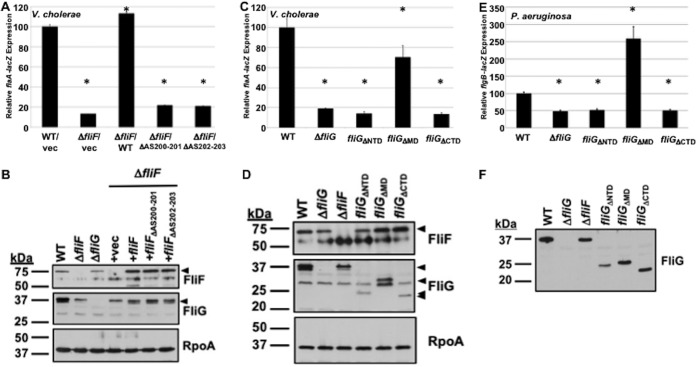
Requirements of V. cholerae and P. aeruginosa for TCS activation and flagellar gene expression. (A) Expression of *flaAp*-*lacZ* transcriptional fusion in WT V. cholerae and isogenic Δ*fliF* mutant. Both the WT and Δ*fliF* mutant strain contained vector alone (vec). The Δ*fliF* mutant also contained vectors to express WT *fliF* (WT) or *fliF* containing in-frame deletions within the ASASVXL motif (depicted as deletion of the AS residues from positions 200 and 201 or positions 202 and 203). (B) Immunoblot analysis of FliF and FliG in whole-cell lysates of WT V. cholerae and isogenic mutants. Specific antiserum to FliF or FliG was used to detect each protein. Detection of RpoA served as a control to ensure equal loading of proteins across strains. (C) Expression of *flaAp*-*lacZ* transcriptional fusion in WT V. cholerae and isogenic *fliG* mutants lacking the N-terminal domain (NTD), middle domain (MD), or C-terminal domain (CTD). (D) Immunoblot analysis of FliF and FliG in whole-cell lysates of WT V. cholerae and isogenic mutants. Specific antiserum to FliF or FliG was used to detect each protein. Detection of RpoA served as a control to ensure equal loading of proteins across strains. (E) Expression of *flgBp*-*lacZ* transcriptional fusion in WT P. aeruginosa and isogenic *fliG* mutants lacking the N-terminal domain (NTD), middle domain (MD), or C-terminal domain (CTD). (F) Immunoblot analysis of FliG in whole-cell lysates of WT P. aeruginosa and isogenic mutants. Specific antiserum to FliG was used to detect the protein. For panels A, C, and E, the level of expression of the transcriptional reporter in each strain is relative to the level of expression in WT V. cholerae or P. aeruginosa, which was set to 100 U. Results from a representative assay, with each sample analyzed in triplicate, are shown. Error bars indicate standard deviations of the average level of expression from three samples. An asterisk indicates increased or decreased reporter activity of a mutant relative to that of the WT strain (*P* < 0.05).

The V. cholerae FliF MS ring protein contains a motif in its periplasmic region (ASASVXL from residues 200 to 206) similar to that of *Salmonella* and C. jejuni FliF. We expressed WT FliF, FliF_ΔAS200-201_, and FliF_ΔAS202-203_ from plasmids in V. cholerae Δ*fliF* and monitored expression of *flaAp*-*lacZ* to assess FlrBC TCS activity. WT FliF restored *flaAp*-*lacZ* expression to the Δ*fliF* mutant, but FliF_ΔAS200-201_ and FliF_ΔAS202-203_ did not (Fig. 5A). Consistent with these observations, WT FliF restored flagellation and motility, but the mutant FliF proteins did not (data not shown). Immunoblot analysis verified that the WT FliF and FliF mutant proteins were produced at similar levels, as were the FliG rotor proteins in these strains (Fig. 5B). Of note, we did observe that FliG is dependent upon FliF for stability, as FliG levels were reduced in the V. cholerae Δ*fliF* mutant with or without empty vector (Fig. 5B). If the ASVXV motif in V. cholerae FliF is required for recruitment to the fT3SS core and/or FliF multimerization into the MS ring around the fT3SS core, as in other flagellates, these data, along with our analysis of mutants lacking FliF, FliG, and fT3SS core proteins, support a model that the formation of the V. cholerae MS ring-rotor-T3SS complex, rather than the production of unassembled fT3SS, MS ring, and rotor proteins, is a cue and regulatory checkpoint influencing FlrBC activity. We were unable to perform a similar analysis in P. aeruginosa, as we could not construct mutations in the FliF ASVXV motif.

We next explored whether differences in the requirements of FliG rotor domains for activity of the V. cholerae, P. aeruginosa, and C. jejuni flagellum-associated TCSs existed. Typical FliG proteins possess three major domains: an N-terminal domain (NTD) that interacts with the cytoplasmic C-terminal domain (CTD) of FliF and is required for multimerization of the MS ring and rotor; a middle domain (MD) to interact with FliM for assembly of the switch complex and the lower portion of the C ring; and a CTD to interact with stator proteins that generate torque for flagellar rotation (62–64). In C. jejuni, only the FliG NTD was required with FliF to form a signal detected by the FlgSR TCS for flagellar rod and hook gene expression (32). We generated V. cholerae and P. aeruginosa *fliG* mutants with deletions of the NTD, MD, or CTD and then assessed TCS- and σ^54^-dependent flagellar gene expression.

FliG mutants lacking the NTD or CTD were unable to restore FlrBC- and σ^54^-dependent flagellar gene expression in V. cholerae (Fig. 5C). However, these proteins were produced at reduced levels compared to those of WT FliG (Fig. 5D), tempering interpretations that these domains are essential for FlrBC TCS activity. In contrast, the expression of FliG_ΔMD_ restored gene expression to ∼70% of the WT level (Fig. 5C), suggesting that at least the middle domain of FliG is not required for FlrBC TCS-dependent flagellar gene expression.

For analysis of P. aeruginosa FliG, WT *fliG* was replaced on the chromosome with *fliG* mutants encoding in-frame deletions of the NTD, MD, or CTD. FliG levels were reduced only modestly with removal of the NTD, but levels of FliG_ΔMD_ and FliG_ΔCTD_ were comparable to those of WT FliG (Fig. 5F). Expression of the FleSR- and σ^54^-dependent *flgB*-*lacZ* reporter was reduced to the same level in the *fliG*_ΔNTD_ and *fliG*_ΔCTD_ mutants as in the Δ*fliG* mutant (Fig. 5E). However, a 2.5-fold increase in P. aeruginosa FleSR activity and flagellar gene expression was observed with FliG_ΔMD_. These observations suggest that at least the P. aeruginosa FliG CTD is required for FleSR TCS activity, whereas the requirement for the NTD is less clear. Considering that only the C. jejuni FliG NTD was required to activate flagellar gene expression (32), our findings indicated different FliG domains, along with FliF, are required in P. aeruginosa (and possibly V. cholerae) for flagellum-associated TCS activity. While we did not analyze MS ring-rotor-fT3SS complex formation directly in V. cholerae and P. aeruginosa, these differences in FliF and FliG domains required for activity of the flagellar TCSs suggest requirements for the assembly of functional fT3SSs vary in these organisms. Additionally, the different sensor domains within FleS and FlrB relative to C. jejuni FlgS may be needed to detect the distinctive signal composed by the different FliF and FliG domains from the respective bacteria.

### A functional link between the polar flagellar transcriptional program and the FlhF/FlhG flagellar biogenesis regulatory system.

Our analysis presented above suggested a connection with many polar flagellates possessing (i) a FlhF/FlhG regulatory system, for spatial and numerical control of polar flagellar biogenesis; (ii) a flagellum-associated TCS whose activity is dependent on MS ring, rotor, and fT3SS proteins; and (iii) a polar flagellar transcriptional program that requires MS ring-rotor-fT3SS protein production and possibly assembly for subsequent flagellar rod and hook gene expression. The FlhF/FlhG flagellar biogenesis regulatory systems and flagellum-associated TCSs are absent from peritrichous bacteria (with Bacillus subtilis as an exception for FlhF and FlhG). In these peritrichous bacteria, a master transcriptional regulator promotes the peritrichous flagellar transcriptional program by expressing MS ring, C ring, rod, and hook genes simultaneously to result in efficient creation of multiple flagella across the surface (Fig. S3) (10, 12, 14, 48). The cooccurrence of the FlhF/FlhG system, flagellum-associated TCSs, and a transcriptional program that separates MS ring-C ring-fT3SS complex gene expression from that of rod and hook genes raises interesting questions. (i) Do polar flagellates require the specific polar flagellar transcriptional program to build flagella in general or to specifically construct polar flagella? (ii) Is ordering rod and hook gene transcription after MS ring-C ring-fT3SS assembly required for an FlhF/FlhG-dependent activity for flagellation? (iii) Can polar flagellates produce flagella (polar or otherwise) if reprogrammed to transcribe flagellar genes similarly to a peritrichous organism in the presence or absence of the FlhF/FlhG flagellar biogenesis regulatory system?

10.1128/mBio.03107-19.6 FIG S3 Construction of V. cholerae mutants for altering a flagellar transcriptional program. The normal peritrichous flagellar transcriptional program for *Salmonella* (left) and the normal polar flagellar transcriptional program for WT V. cholerae (middle) are shown. Note the WT V. cholerae polar flagellar transcriptional program (middle) includes both FlrA-dependent transcription of initial flagellar genes, the regulatory checkpoint associated with MS ring, rotor, and fT3SS core proteins discovered in this work, and FlrBC- and σ^54^-dependent transcription of flagellar rod and hook genes. Full V. cholerae operons for FlrA- or FlrBC-/σ^54^-dependent operons are shown. For creation of transcriptional reprogramming mutants (right), the native σ^54^- and FlrBC TCS-dependent promoters for the *flgB*, *flgF*, and *flgK* operons encoding flagellar rod and hook genes were replaced with the FlrA-dependent *fliE* promoter (*fliEp*). Promoter mutations were made individually or in different combinations to create a full array of V. cholerae mutants with up to all three flagellar rod and hook operons dependent on FlrA for transcription (right), rather than on FlrBC TCS and σ^54^ (middle), to resemble a peritrichous flagellar transcriptional program (left). Download FIG S3, TIF file, 1.1 MB. Copyright © 2020 Burnham et al. 2020 Burnham et al. This content is distributed under the terms of the Creative Commons Attribution 4.0 International license.

For these analyses, we developed a transcriptional reprogramming strategy in V. cholerae so that expression of one, two, or all three FlrBC- and σ^54^-dependent flagellar rod and hook operons (*flgBCDE*, *flgFGHIJ*, and *flgKL*) were under the control of the FlrA master transcriptional regulator that normally only controls expression of MS ring, C ring, fT3SS, and FlrBC TCS genes (Fig. S3). By replacing the FlrBC- and σ^54^-dependent *flgB*, *flgF*, and *flgK* promoters with the FlrA-dependent *fliE* promoter at the native locations on the V. cholerae chromosome (Fig. S3) (18), the requirement to detect the regulatory checkpoint centered around MS ring-rotor-fT3SS protein production for rod and hook gene expression would be bypassed. Thus, V. cholerae would produce some or all rod and hook proteins earlier than normal and at the same time as the MS ring, C ring, and fT3SS proteins, which shifts the normal V. cholerae polar flagellar transcriptional program to one more closely following the peritrichous transcriptional program that normally exists in E. coli and *Salmonella* species (Fig. S3) (10, 15).

In these experiments, we retained *flaA* expression under the control of its natural FlrBC- and σ^54^-dependent promoter. By doing so, the FlaA major flagellin in these V. cholerae transcriptional reprogramming mutants was produced either simultaneously with some rod and hook proteins as normal (if the mutant contained only one or two promoter alterations) or after all rod and hook proteins (if the mutant contained all three promoter alterations) (Fig. S3). Transcription of *flaA* after rod and hook protein production in these V. cholerae mutants would be temporally similar to how most polar and peritrichous flagellates naturally express major flagellins from a σ^28^-dependent promoter after formation of the flagellar rod and hook (Fig. 1 and Fig. S3). As shown below, maintaining *flaA* expression under its natural FlrBC- and σ^54^-dependent promoter allowed for sufficient flagellin production for filament assembly and motility in many transcriptionally reprogrammed mutants.

We also deleted *flhF* and *flhG* from these V. cholerae transcriptional reprogramming mutants to examine any potential link between the activity of the FlhF/FlhG regulatory system for monotrichous flagellation in V. cholerae and alteration in the timing of rod and hook gene transcription relative to MS ring, C ring, and fT3SS expression. As reported previously, FlhF is required for flagellar biogenesis in V. cholerae, and we confirmed that V. cholerae Δ*flhF* lacked flagella (7 and data not shown); thus, we could not analyze flagellation in our transcriptional reprogramming mutants in the Δ*flhF* background. However, deletion of *flhG* from the classical V. cholerae O395 strain allowed for the production of polar flagella but with hyperflagellation due to the lack of proper numerical control of flagellar biogenesis (7). Hyperflagellation was proposed to be due to increased *flrA* expression to cause overexpression of all flagellar genes, resulting in multiple flagella. However, this hyperflagellated phenotype was unstable in V. cholerae O395 Δ*flhG*; after subsequent *in vitro* passaging, flagellar gene expression was reduced and the monotrichous phenotype returned (7).

**FIG 6:**
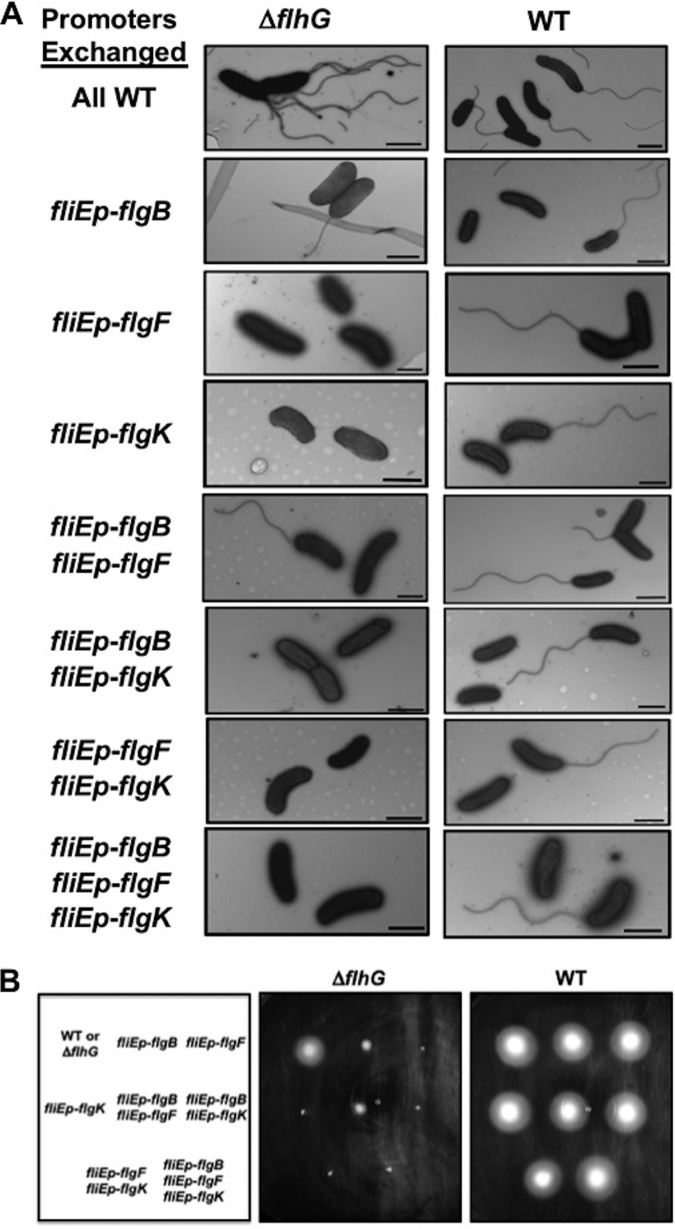
Effect of transcriptional reprogramming of flagellar genes on V. cholerae flagellation. Shown are electron micrographs (A) and motility phenotypes (B) of WT V. cholerae, the V. cholerae Δ*flhG* mutant, or isogenic transcriptional reprogramming mutants in which one or more operons with an FlrBC TCS- and σ^54^-dependent promoter was replaced with the *fliE* promoter (*fliEp*), as indicated, to transition flagellar gene transcription toward the peritrichous flagellar program. All combinations of transcriptional reprogramming mutants were made in V. cholerae Δ*flhG* and WT V. cholerae strains (left and right columns, respectively). In panel A, the bar represents 1 μm. In panel B, motility was assessed after inoculating overnight cultures in LB with 0.3% agar and incubation at 37°C for 8 h. The box on the left in panel B is a map depicting how the Δ*flhG* or WT strain and their corresponding transcriptionally reprogrammed mutants were inoculated into motility agar in the center and right panels.

**FIG 7:**
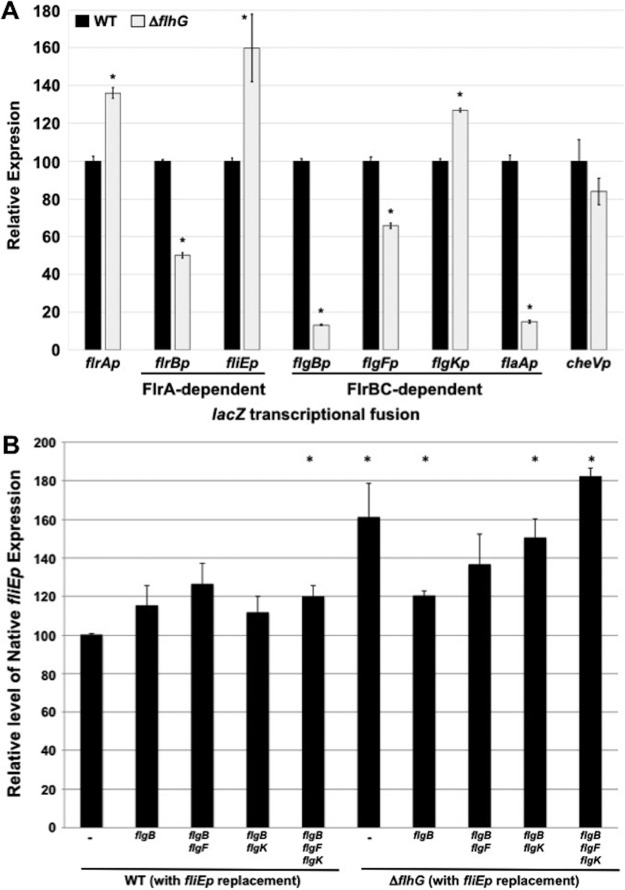
Activity of flagellar promoters in WT V. cholerae and Δ*flhG* mutant strains. (A) Expression of *lacZ* transcriptional fusions from different flagellar promoters in WT V. cholerae C6706 and an isogenic Δ*flhG* mutant. *flrAp-, flrBp-, fliEp-, flgBp-, flgF-, flgKp-, flaAp-,* and *cheVp*-*lacZ* transcriptional reporters were maintained on plasmids in V. cholerae strains. The level of expression of each transcriptional reporter in each mutant is relative to the level of expression in WT V. cholerae, which was set to 100 U. Results from a representative assay with each sample analyzed in triplicate are shown. Error bars indicate standard deviations of the average level of expression from three samples. An asterisk indicates significant difference in expression from the WT containing vector alone (*P* < 0.05). (B) *fliE* promoter activity in WT V. cholerae and the Δ*flhG* mutant with either a normal polar flagellar transcriptional program or transcriptionally reprogrammed toward a peritrichous pattern. An *fliEp*-*lacZ* transcriptional reporter was introduced on a plasmid in WT V. cholerae or the Δ*flhG* mutant with a normal polar flagellar transcriptional program (indicated by a dash) and select transcriptional reprogramming mutants. In the transcriptional reprogramming mutants analyzed, the promoter for one or more rod and hook operons that was replaced with the *fliE* promoter is indicated. The level of expression of the *fliEp*-*lacZ* transcriptional reporter in each mutant is relative to the level of expression in WT V. cholerae, which was set to 100 U. Results from a representative assay, with each sample analyzed in triplicate, are shown. Error bars indicate standard deviations of the average level of expression from three samples. An asterisk indicates significant difference in expression relative to that of WT V. cholerae (*P* < 0.05).

We verified the hyperflagellation phenotype of a Δ*flhG* mutant in V. cholerae C6706 (the strain used throughout this study). In our analysis, 54% of the WT population produced exclusively monotrichous flagella, with 46% lacking a flagellum (Table 1 and Fig. 6A). Only a small minority of the WT flagellated population was hyperflagellated (1.4%). V. cholerae Δ*flhG* cells produced flagella in a higher percentage of the population (67.5% versus 54.2% for the WT). Furthermore, 61% of flagellated Δ*flhG* cells were hyperflagellated by producing 2 to 7 polar flagella at a single pole (Table 1 and Fig. 6A). The flagella of V. cholerae Δ*flhG* cells occasionally appeared thinner in structure with possible defects in flagellar sheath formation compared to the monotrichous flagellum of WT V. cholerae. Despite hyperflagellation, the Δ*flhG* mutant was motile, although modestly less so than the WT (Fig. 6B). Contrary to a previous report, hyperflagellation was stable in the V. cholerae C6706 Δ*flhG* strain (7). Expression of *lacZ* transcriptional reporters linked to promoters from different classes of flagellar genes were generally altered in the Δ*flhG* strain but with either increased or decreased expression depending on the promoter examined (Fig. 7A). Thus, we could not link hyperflagellation to gross overexpression of flagellar genes in the Δ*flhG* strain, as previously hypothesized (7), suggesting that FlhG regulates flagellar number by another means, such as controlling *in vivo* FlhF activity by modulating its GTP-binding state, as postulated for Vibrio alginolyticus and C. jejuni (3–5). In summary, the V. cholerae Δ*flhG* mutant with a WT polar flagellar transcriptional program efficiently produced polar flagella (possibly due to dysregulated FlhF activity), as observed by an increase in polarly flagellated cells and in the number of polar flagella per cell (hyperflagellation).

**TABLE 1:** Measurement of flagellation in WT V. cholerae, V. cholerae Δ*flhG* mutant, and transcriptional reprogramming mutants

Strain	% Of population^*a*^		% Hyperflagellated	Strain	% Of population^*a*^		% Hyperflagellated^*b*^
	Flagellated	Aflagellated			Flagellated	Aflagellated	
WT	54.2 ± 2.5	45.9 ± 2.5	1.4 ± 2.5	Δ*flhG*	67.5 ± 11.1	32.5 ± 11.1	61.4 ± 11.6
*fliEp*-*flgB* operon	56.6 ± 4.3	43.4 ± 4.3	0 ± 0	Δ*flhG fliEp*-*flgB* operon	8.3 ± 4.1^*c*^	91.7 ± 4.1^*c*^	0 ± 0^*c*^
*fliEp-flgF* operon	48.2 ± 4.0	51.8 ± 4.0	0.8 ± 1.3	Δ*flhG fliEp-flgF* operon	0 ± 0^*c*^	100 ± 0^*c*^	0 ± 0^*c*^
*fliEp-flgK* operon	53.4 ± 5.6	46.6 ± 5.6	0.9 ± 0.9	Δ*flhG fliEp-flgK* operon	0 ± 0^*c*^	100 ± 0^*c*^	0 ± 0^*c*^
*fliEp-flgB* operon	48.1 ± 6.3	51.9 ± 6.3	0.6 ± 0.5	Δ*flhG fliEp*-*flgB* operon	13.9 ± 2.1^*c*^	86.1 ± 2.1^*c*^	9.5 ± 4.8^*c*^
*fliEp-flgF* operon				*fliEp-flgF* operon			
*fliEp*-*flgB* operon	55.6 ± 2.0	44.4 ± 2.0	2.0 ± 0.6	Δ*flhG fliEp*-*flgB* operon	0 ± 0^*c*^	100 ± 0^*c*^	0 ± 0^*c*^
*fliEp-flgK* operon				*fliEp-flgK* operon			
*fliEp-flgF* operon	50.9 ± 6.4	49.1 ± 6.4	4.4 ± 6.3	Δ*flhG fliEp-flgF* operon	0.4 ± 0.6^*c*^	99.6 ± 0.6^*c*^	0 ± 0^*c*^
*fliEp-flgK* operon				*fliEp-flgK* operon			
*fliEp-flgB* operon	53.0 ± 3.5	47.0 ± 3.5	2.1 ± 1.9	Δ*flhG fliEp*-*flgB* operon	0 ± 0^*c*^	100 ± 0^*c*^	0 ± 0^*c*^
*fliEp-flgF* operon				*fliEp-flgF* operon			
*fliEp-flgK* operon				*fliEp-flgK* operon			

a For each strain, three independent samples with at least 100 bacterial cells per sample were analyzed. Values are presented as averages ± standard deviations.

b The values for the hyperflagellated population represent the percentages of the flagellated population that had at least two or more flagella at one pole of the cell.

c The level of flagellation, aflagellation, or hyperflagellation of individual V. cholerae Δ*flhG* transcriptional reprogramming mutants was statistically significantly different from the V. cholerae Δ*flhG* mutant with normal flagellar gene transcription (*P* < 0.05). No significant differences were noted among WT V. cholerae and the respective transcriptional reprogramming mutants.

Upon alteration of the polar flagellar transcriptional program in the V. cholerae Δ*flhG* mutant to more closely resemble a peritrichous transcriptional program by replacing the FlrBC- and σ^54^-dependent promoter for a single rod and hook operon with the FlrA-dependent *fliE* promoter (Fig. S3), we observed almost complete elimination of polar flagellar biogenesis and the prominent Δ*flhG* hyperflagellation phenotype (Table 1 and Fig. 6A). Further shifts toward the peritrichous transcriptional program in which two or all three rod and hook promoters were replaced with *fliEp* were also mostly or completely aflagellated. The only Δ*flhG* transcriptional reprogramming mutants that were flagellated were those with *fliEp* expressing the *flgB* operon alone or both the *flgB* and *flgF* operons, but a significantly smaller population of cells were flagellated than those of the Δ*flhG* mutant with the normal polar flagellar transcriptional program (8.3% to 13.9%) (Table 1 and Fig. 6A). These mutants also were severely reduced for motility relative to V. cholerae WT and Δ*flhG* strains (Fig. 6B). The flagella of the Δ*flhG fliEp*-*flgBCDE* mutant that were produced tended to be shorter than those of WT V. cholerae and the Δ*flhG* mutant (Fig. 6A).

In select Δ*flhG* transcriptional reprogramming mutants, we did not observe gross decreases in FlrA activity, which was driving rod and hook gene expression in these mutants, as monitored by *fliEp*-*lacZ* expression relative to that of WT V. cholerae (Fig. 7B). Instead, FlrA activity was comparable to or modestly greater in Δ*flhG* than in WT strains. Thus, reduced or absent flagellation in the Δ*flhG* transcriptional reprogramming mutants was not due to impaired FlrA activity and expression of rod and hook genes from the *fliE* promoter. Our data indicate that polar flagellar biogenesis efficiently occurs in V. cholerae with altered activity of the FlhF/FlhG flagellar biogenesis system (as in a Δ*flhG* mutant), albeit with hyperflagellation, as long as the WT polar flagellar transcriptional program is maintained by the FlrBC TCS to order rod and hook gene transcription after MS ring-C ring-fT3SS production. Any alteration toward a peritrichous flagellar transcriptional program without an intact FlhF/FlhG polar flagellar regulatory system leads to severe reduction or loss of flagellation.

We next addressed whether flagellar biogenesis was affected in a V. cholerae hybrid that contained an intact FlhF/FlhG flagellar biogenesis regulatory system but with alterations to follow more closely a peritrichous flagellar transcriptional program. Contrary to the V. cholerae Δ*flhG* transcriptional reprogramming mutants, the level of monotrichous flagellation in any population of transcriptional reprogramming mutants with a WT FlhF/FlhG regulatory system did not change relative to that of WT V. cholerae (Table 1 and Fig. 6A), even in the V. cholerae mutant with all three *fliE* promoter substitutions (*fliEp*-*flgB*, *fliEp*-*flgF*, and *fliEp*-*flgK*), to most closely resemble a peritrichous flagellar transcriptional program. We also did not detect differences in flagellar filament length, appearance, or function in motility *in vitro* (Fig. 6A and B). Thus, V. cholerae with a peritrichous flagellar transcriptional program produces polar flagella normally during *in vitro* growth, as long as the FlhF/FlhG flagellar biogenesis regulatory system is fully operational and intact. Disruption of the FlhF/FlhG system in such a transcriptionally reprogrammed V. cholerae cell abolishes or greatly reduces flagellation and motility. Thus, we propose that flagellum-associated TCSs of polar flagellates mediate the polar flagellar transcriptional program, characterized by the ordered transcription of flagellar rod and hook genes after the MS ring-rotor-fT3SS regulatory checkpoint during flagellar assembly, to allow polar flagellates flexibility in producing flagella and retaining motility (even with hyperflagellation) when the FlhF/FlhG system may not function properly.

## DISCUSSION

Polarly flagellated bacteria are present in a wide range of proteobacterial classes. To achieve species-specific flagellation patterns for optimal motility, each polar flagellate must have transcriptional mechanisms to correctly control flagellar gene expression and biogenesis regulators to create specific flagellation patterns composed of the correct number of flagella at one or both poles. However, little is known regarding how broadly conserved these transcriptional and biosynthetic regulatory mechanisms are and how they may be intertwined for correct biogenesis of polar flagella and polar flagellation patterns.

To explore what may contribute to the conserved polar flagellar transcriptional program for creating a specific ordering of transcription of different classes of flagellar genes, we showed by *in silico* analysis that Gram-negative polar flagellates can be divided into two distinct groups. One group produces an FlhF/FlhG flagellar biogenesis regulatory system with a flagellum-associated TCS, and another is composed of alphaproteobacteria that lack an FlhF/FlhG system. We found that FlhF/FlhG- and flagellum-associated TCS-positive polar flagellates may be subdivided into two or more groups based on the sensory domains of the TCS kinases. The different kinases are best represented by C. jejuni FlgS, which contains a predicted coiled-coil domain in the sensory region, and V. cholerae FlrB/P. aeruginosa FleS, which contain PAS domains.

Despite their identification years ago, the actual signals detected by the V. cholerae FlrB and P. aeruginosa FleS sensor kinases had not been analyzed. We previously discovered that the amphitrichous polar flagellate C. jejuni detects a regulatory checkpoint formed by the MS ring and rotor assembling around the fT3SS core by FlgS of its flagellum-associated FlgSR TCS (32). In our current work, we found that MS ring, rotor, and fT3SS proteins are broadly required for the activity of the V. cholerae FlrB and P. aeruginosa FleS sensor kinases to result in rod and hook gene transcription. Additionally, we found that V. cholerae FliF mutants that likely fail to form an MS ring were defective in FlrBC TCS activity. These findings are similar to our previous studies in C. jejuni and what others have reported in H. pylori (32, 40, 41, 43). Furthermore, we accumulated evidence that for at least P. aeruginosa (and possibly V. cholerae), more domains of the FliG rotor were required for flagellum-associated TCS activity than in C. jejuni. These combined data build support for a general conserved mechanism in which flagellum-associated TCSs of polar flagellates broadly detect a regulatory checkpoint centered around MS ring-rotor-fT3SS assembly as a signal to facilitate the polar flagellar transcriptional program that orders rod and hook gene expression as a subsequent step for the progression of flagellar biogenesis. We suspect that the FliF MS ring, FliG rotor, and fT3SS core proteins (FlhA, FlhB, FliP, FliQ, and FliR) would be required for the activity of flagellum-associated TCSs for rod and hook gene expression in many other polar flagellates that have yet to be explored.

A caveat to our work presented here is that we have not yet been able to detect a direct interaction between the FlrB or FleS kinase with the FliF MS ring or FliG rotor proteins, like we observed with C. jejuni FlgS, as a mechanism to monitor formation of the MS ring and rotor around the fT3SS core (32). This indicates that the interactions between the kinases and flagellar proteins are weaker or more transient than in C. jejuni or that FlrB and FleS monitor fT3SS assembly indirectly through an unidentified factor. Differences in FliG domains potentially required for signal formation and detection, the abilities of the kinases to interact with MS ring and rotor components, and potential sensory domains within the kinases may reflect varied mechanisms for how these flagellum-associated TCSs monitor the formation of the regulatory checkpoint. Regardless, our findings continue to support that bacteria have mechanisms to monitor the formation of intracellular macromolecular structures and to relay information that influences behavior. Verification and deeper investigation of how each flagellum-associated TCS might detect MS ring-rotor-fT3SS assembly will be insightful for how these regulatory systems function.

When also considering the alphaproteobacterial polar flagellates that lack the FlhF/FlhG flagellar biogenesis regulatory system, monitoring formation of a competent fT3SS by different systems to influence subsequent rod and hook gene expression emerges as a common strategy across Gram-negative polar flagellates for the development of the conserved polar flagellar transcriptional program. Of the alphaproteobacterial polar flagellates in the reference collection, the flagellar system of C. crescentus is the best characterized (reviewed in references 65). C. crescentus executes the polar flagellar transcriptional program so that transcription of MS ring, C ring, and fT3SS genes occurs prior to σ^54^-dependent transcription of rod and hook genes (66). C. crescentus σ^54^ requires FlbD, an enhancer-binding protein similar to the C. jejuni FlgR, V. cholerae FlrC, and P. aeruginosa FleR response regulators of the flagellum-associated TCSs, to activate flagellar rod and hook gene expression (67–71). MS ring, C ring, and fT3SS proteins are also required for FlbD activity and FlbD- and σ^54^-dependent rod and hook gene expression (69, 71). Thus, FlbD activity is linked to MS ring-C ring-fT3SS complex formation, yet FlbD lacks a cognate sensor kinase like FlgS, FlrB, or FleS to monitor flagellar assembly and control its activity. The FliX transactivating factor has been identified as the link that relays the status of fT3SS assembly to positively or negatively control the activity of FlbD to bind to target flagellar rod and hook promoters (72–75). Although its regulatory mechanism is not understood, FliX does not function as a kinase to transduce signals regarding fT3SS assembly.

As diverse polar flagellates have evolved different mechanisms to create and maintain the polar flagellar transcriptional program so that rod and hook gene expression occurs after formation of the regulatory checkpoint at fT3SS assembly, we hypothesized that this program is beneficial for biogenesis of polar flagella. E. coli and *Salmonella*, as models with the peritrichous flagellar transcriptional program, do not recognize this checkpoint and transcribe most basal, rod, and hook genes simultaneously to efficiently build peritrichous flagella (10, 14, 48). Since, in this study, we show a V. cholerae cell containing the WT FlhF/FlhG regulatory system engineered with a peritrichous flagellar transcriptional program produced a monotrichous flagellum efficiently, the type of flagellar transcriptional program itself does not seem to determine the peritrichous or polar flagellation pattern of the species.

Instead, we discovered that possessing a polar transcriptional program and the FlhF/FlhG flagellar biogenesis regulatory system allows polar flagellation while retaining motility to a modest extent when perturbations to FlhF and FlhG activity occur. For example, the V. cholerae Δ*flhG* mutant with a polar flagellar transcriptional program was hyperflagellated, indicating high proficiency in producing flagella. However, the Δ*flhG* mutant was less motile than the WT monotrichous strain, likely due to the inability of the Δ*flhG* mutant to coordinate multiple rotating polar flagella for optimal swimming motility. In contrast, flagellation was severely diminished or even abolished in V. cholerae mutants that more closely resembled most peritrichous bacteria by lacking a properly functioning FlhF/FlhG flagellar biogenesis system (through deletion of *flhG*) and engineered with a peritrichous flagellar transcriptional program. Thus, the peritrichous transcriptional program is much more affected by alterations in FlhF/FlhG activity in a polar flagellate, resulting in greatly decreased flagellation, motility, and, likely, fitness in nature. Currently, it is unknown whether FlhF/FlhG activity is naturally regulated or altered by extrinsic factors or metabolic capacity. We have observed hyperflagellation in WT polarly flagellated systems in a small minority of cells (in V. cholerae in this work and previously in C. jejuni [3, 76]). Thus, the FlhF/FlhG flagellar biogenesis regulatory system is likely affected by stochastic influences on a cell-to-cell basis that may at least alter FlhG activity and likely its ability to regulate FlhF. It remains to be determined whether the polar flagellar transcriptional program also provides an advantage to C. crescentus that has a different collection of determinants to produce polar flagella during developmental stages and asymmetrical division (77–80).

Our analysis of V. cholerae flagellar transcriptional reprogramming mutants provided some intriguing observations, but many questions remain. One such question is what advantage exactly does the polar flagellar transcriptional program provide for the FlhF/FlhG regulatory system to enable efficient flagellation when FlhF or FlhG activity is altered that the peritrichous flagellar transcriptional program does not provide. It is currently unclear how FlhG controls flagellum numbers in V. cholerae. FlhG orthologs regulate flagellum number by at least two different processes, including influencing the activity of FlhF or a master regulator of flagellar gene transcription, such as FlrA (1–5, 7–9, 81). In contrast to a previous report, we did not observe a broad increase in transcription across classes of flagellar genes in the V. cholerae Δ*flhG* mutant that would explain the consistent hyperflagellation phenotype we observed (7). Thus, hyperflagellation in the V. cholerae Δ*flhG* mutant may be due to a dysregulated, hyperactive FlhF, which has been proposed in C. jejuni and other *Vibrio* species (3–5).

The molecular mechanism by which the FlhF GTPase influences polar flagellation has not been determined in many bacteria. One hypothesis includes that FlhF localizes MS ring, C ring, and fT3SS core proteins at a pole or facilitates interactions between these proteins to create a new flagellum (1, 2, 82). FlhG presumably functions in some polar flagellates to transition FlhF from a GTP-bound “on” state competent for a function to initiate flagellation to a GDP-bound “off” state. Tight control of FlhF by FlhG may be required so that FlhF can organize flagellar proteins properly for MS ring-C ring-fT3SS assembly either with the stepwise production of MS ring, C ring, fT3SS, rod, and hook proteins provided by the polar flagellar transcriptional program or with their simultaneous production facilitated by the peritrichous flagellar transcriptional program. However, a dysregulated FlhF in the Δ*flhG* mutant may be unable to perform its natural function in flagellation when MS ring, C ring, fT3SS, rod, and hook proteins are produced at the same time. Consistent with this, we observed a great reduction or abolishment of flagellation when some or all rod and hook proteins were simultaneously produced with MS ring, C ring, and fT3SS proteins in the Δ*flhG* mutant engineered to follow more closely a peritrichous transcriptional flagellar program. Our findings may point toward a more expansive role for FlhF: in addition to its hypothesized role in assisting polar assembly of the MS ring-C ring-fT3SS complex, FlhF may also organize flagellar proteins, such as the rod and hook proteins, for secretion via the fT3SS. If so, production of multiple FlhF-interacting proteins (fT3SS complex proteins and their secretion substrates) simultaneously may overwhelm a dysregulated FlhF so that flagellar biogenesis does not occur. Other possibilities exist, including that the peritrichous flagellar transcriptional program in a Δ*flhG* mutant disrupts flagellar protein stoichiometry. In this case, there may not be enough rod and hook proteins produced for the multiple fT3SSs that may form in the Δ*flhG* mutant. Undoubtedly, there are functions for FlhF and FlhG that are not yet adequately understood to reveal how the polar flagellar transcriptional program contributes to the FlhF/FlhG flagellar biogenesis system for efficient polar biogenesis. Continued exploration will likely further reveal how transcriptional and biosynthetic processes are integrated in polar flagellates to construct the ideal number and positioning of these macromolecular machines for motility in bacterial cells.

Our findings raise some questions regarding how different flagellar transcriptional programs formed across flagellated species. One prominent question is whether polar and peritrichous flagellar transcriptional programs developed independently of each other or if one evolved from the progenitor of another. It is clear that flagellar structural components are largely conserved across bacterial species. Even the mechanism to detect rod and hook formation as a late regulatory checkpoint required for activation of σ^28^ and expression of terminal flagellar genes is widely conserved (11, 13, 30). However, regulatory factors and mechanisms required for expression of flagellar components required for formation of the fT3SS, rod, and hook differ in peritrichous and polar flagellates. Most peritrichous bacteria (albeit with B. subtilis as a Gram-positive exception) have a seemingly less complex flagellar transcriptional program so that MS ring, C ring, fT3SS core, rod, and hook proteins are produced simultaneously by the activity of a flagellar master regulator, and these bacteria do not require FlhF or FlhG to efficiently construct multiple flagella across their surfaces.

Polar flagellates may have originated from a peritrichous progenitor but also could have developed independently. Comparisons between C. crescentus and many other Gram-negative polar flagellates as discussed above clearly show that different polar flagellar biogenesis systems exist in the presence of somewhat conserved regulatory mechanisms to facilitate the polar flagellar transcriptional program, indicating convergent evolution of polar flagellates. Regardless, our findings suggest that a species needs to acquire a polar flagellar biogenesis system (such as the FlhF/FlhG system) and a mechanism to order flagellar genes for the polar flagellar transcriptional program (such as a flagellum-associated TCS) to become an efficient polar flagellate. Possessing only the FlhF/FlhG system with the peritrichous program does not guarantee optimal flagellation and motility if FlhF/FlhG activity is affected by extrinsic, stochastic factors.

It is unknown which came first in a polar flagellate, the FlhF/FlhG polar flagellar biogenesis system or the flagellum-associated TCSs, to drive the polar flagellar transcriptional program. Both the FlhF GTPase and FlhG ATPase are members of the SIMIBI class of nucleotide-binding proteins that commonly function in cellular organization and protein targeting (83, 84). FlhF is related to the Ffh GTPase of the signal recognition particle system, whereas FlhG is closely associated with the MinD and ParA ATPases that generally perform partitioning functions related to division and DNA segregation (6, 83–85). Development of the FlhF/FlhG flagellar biogenesis regulatory system, perhaps from Ffh and MinD/ParA superfamilies, could have caused the emergence of a polar flagellate in a Gram-negative organism. The motile, monotrichous V. cholerae strain we engineered with an intact FlhF/FlhG system and a peritrichous flagellar transcriptional program might resemble this ancestor. As revealed in this work, this bacterium is heavily reliant on a precisely functioning FlhF/FlhG system to form any flagella and retain some level of motility; perturbations to FlhF or FlhG activity severely reduce or completely abolish flagellation. By possessing a mechanism mediated by the flagellum-associated TCSs to order rod and hook gene transcription after production of MS ring, rotor, and fT3SS proteins (and possibly assembly of a functional fT3SS), a bacterium can produce polar flagella with some alterations to FlhF/FlhG activity. In this bacterium, an optimally functioning FlhF/FlhG system allows for the correct number and placement of polar flagella and WT motility; an impaired FlhF/FlhG system (at least by altering FlhG) results in polar flagellation with extra flagella produced and at least modest motility. This hyperflagellated bacterium has an advantage over one with the FlhF/FlhG system and a peritrichous program that cannot maintain flagellation and motility with perturbations to the FlhF/FlhG system.

Modulations in FlhF and FlhG activity in different species with flagellum-associated TCSs to maintain the polar flagellar transcriptional program and flagellar biogenesis may have facilitated the emergence of different polar flagellation patterns, amphitrichous, lophotrichous, and monotrichous. An example of this is C. jejuni and H. pylori, which, while closely related, produce amphitrichous and lophotrichous flagella, respectively, yet have the FlhF/FlhG flagellar biogenesis regulatory system and similar flagellum-associated FlgSR TCSs. A study comparing FlhF and FlhG biochemical activity and biological function between these two bacterial species has not been conducted. Although many details remain to be discovered for how FlhF and FlhG function in polar flagellates, our results indicate regulatory links between the FlhF/FlhG flagellar biogenesis regulatory systems and the order of flagellar protein production controlled by the flagellum-associated TCSs for polar flagellar biogenesis.

## MATERIALS AND METHODS

### General growth and storage conditions of bacteria.

C. jejuni 81-176 strains were stored at −80°C as frozen stocks in a solution of 85% Mueller-Hinton (MH) broth and 15% glycerol. All C. jejuni strains were grown from frozen stocks on MH agar for 48 h under microaerobic conditions (10% CO_2_, 5% O_2_, and 85% N_2_) at 37°C and then restreaked on MH agar and grown for another 16 h under microaerobic conditions at 37°C. As required, antibiotics were added to MH medium at the following concentrations: 10 μg/ml trimethoprim (TMP), 15 μg/ml chloramphenicol, 50 or 100 μg/ml kanamycin, or 0.5, 1, 2, or 5 mg/ml streptomycin.

V. cholerae C6706 *lacZ*, a spontaneous *lacZ* derivative of the WT El Tor C6706 strain, and isogenic mutants were used for all analyses involving V. cholerae strains (86). Pseudomonas aeruginosa PA14 and isogenic mutants were used for all analyses involving P. aeruginosa strains (87, 88). E. coli, V. cholerae, and P. aeruginosa strains were routinely grown in LB broth at 37 or 30°C and stored as frozen stocks at −80°C in a solution of 80% LB and 20% glycerol. As required, antibiotics or growth inhibitors were added to LB broth or agar at the following concentrations: 100 μg/ml ampicillin, 10 μg/ml chloramphenicol, 100 μg/ml kanamycin, 100 μg/ml streptomycin, 15 μg/ml gentamicin, 12.5 μg/ml tetracycline, and 10% sucrose.

### Bacterial strains and plasmid construction.

All methodologies to construct plasmids and C. jejuni, V. cholerae, and P. aeruginosa mutants are described in Text S1 of the supplemental material. All bacterial strains and plasmids used in this study are listed in Tables S1 and S2.

10.1128/mBio.03107-19.1 TEXT S1 Additional materials and methods. Download Text S1, PDF file, 0.2 MB. Copyright © 2020 Burnham et al. 2020 Burnham et al. This content is distributed under the terms of the Creative Commons Attribution 4.0 International license.

10.1128/mBio.03107-19.2 TABLE S1 Bacterial strains used in this study. Download Table S1, PDF file, 0.2 MB. Copyright © 2020 Burnham et al. 2020 Burnham et al. This content is distributed under the terms of the Creative Commons Attribution 4.0 International license.

10.1128/mBio.03107-19.3 TABLE S2 Plasmids used in this study. Download Table S2, PDF file, 0.1 MB. Copyright © 2020 Burnham et al. 2020 Burnham et al. This content is distributed under the terms of the Creative Commons Attribution 4.0 International license.

### Bioinformatic analyses.

Complete reference bacterial genomes were acquired from www.ncbi.nlm.nih.gov/assembly to form a database containing 117 genomes. tBLASTn was run against the genome database to identify the top-scoring hit for each genome in the database for the following protein sequences in FASTA format from the UniProt database: E. coli FlgH (P0A6S0), V. cholerae FlhF (C3LP19) or C. jejuni FlhF (A0A0H3P9N0), and V. cholerae FlrB (C3LPE1) or C. jejuni FlgS (A0A0H3PDD6). To perform a reciprocal best hit sequence alignment, we performed another tBLASTn search with each top-scoring hit from each genome against the genome containing each protein query and only considered positive hits as those that were able to identify the protein query as the top-scoring hit in the respective genome. We additionally acquired 51 sensor histidine kinase sequences from the V. cholerae N16961 proteome from UniProt. We performed reciprocal best hit sequence alignments against the reference bacterial genome database with these 51 V. cholerae sensor kinases and calculated the Pearson correlation coefficient between each of the 51 sets of sensor kinase hits and V. cholerae FlhF hits in the reference bacterial genomes. For completion of all bioinformatics analysis, the following software was used: tBLASTn (www.ncbi.nlm.nih.gov) for reciprocal best hit sequence alignments, Biopython (www.biopython.org) to read XML files and prepare command lines, Python 2.7.6 (www.python.org) to run scripts, and NumPy (www.numpy.org) to calculate Pearson correlation coefficients.

### Arylsulfatase assays.

Arylsulfatase assays were used to measure the level of expression of the *flaB*::*astA* transcriptional fusion on the chromosome of C. jejuni Δ*astA* strains as previously described (31, 89, 90). Each strain was analyzed in triplicate, and each assay was performed three times. The level of expression of the transcriptional fusion in each strain was calculated relative to the expression in the wild-type C. jejuni Δ*astA* strain, which was set to 100 U.

### β-Galactosidase assays.

The level of gene expression in V. cholerae and P. aeruginosa strains was compared by monitoring the β-galactosidase activity of strains harboring *lacZ* transcriptional fusions to specific promoters by standard procedures (91). Strains were grown in LB at 37°C with shaking to an optical density at 600 nm (OD_600_) of approximately 0.8 prior to the start of the assays. Each strain was analyzed in triplicate, and each assay was performed three times. The level of expression of the transcriptional fusion in each strain was calculated relative to the expression in wild-type V. cholerae C6706 *lacZ* or PA14, which was set to 100 U.

### Antiserum production.

All use of animals in experimentation has been approved by the IACUC at the University of Texas Southwestern Medical Center. Recombinant protein for antiserum production was produced by first cloning the coding sequences from codon 2 to the stop codon of V. cholerae *flrB*, *fliG*, and *rpoA* into the SmaI site, the BamHI site, or the BamHI and SalI sites of pGEX4T-2 to create N-terminal fusions of glutathione *S*-transferase. For recombinant V. cholerae FliF, the region of *fliF* encoding the predicted periplasmic domain, from codons 45 to 473, was cloned into the BamHI and SalI sites of pQE30 to create an N-terminal fusion of 6× His tag. For recombinant P. aeruginosa FliG, the coding sequence from codon 2 to the stop codon was cloned into the BamHI and SmaI sites of pQE30 to create an N-terminal fusion of a 6× His tag. Resultant plasmids were transformed into BL21(DE3) or XL1-Blue and then induced in LB broth with 1 mM isopropyl-β-d-thiogalactopyranoside (IPTG). Recombinant protein was purified from the soluble fractions by affinity chromatography according to the manufacturer’s instructions. Purified recombinant protein was used to immunize guinea pigs by standard procedures for antiserum generation via a commercial vendor (Cocalico Biologicals).

### Immunoblot analysis.

Whole-cell lysates (WCLs) of V. cholerae and P. aeruginosa strains for immunoblot analysis were prepared by first inoculating 5 ml LB with a 1:50 dilution of overnight cultures. Cultures were grown at 37°C with shaking to an OD_600_ of 0.8. One-milliliter aliquots of each culture were recovered by centrifugation in microcentrifuge tubes, washed once with phosphate-buffered saline (PBS), and then resuspended in 50 μl of 1× SDS-loading buffer. Samples were boiled for 5 min prior to separation by SDS-PAGE and transferred to membranes for immunoblotting by standard procedures. For specific detection of proteins in WCLs, 10 μl of WCLs was analyzed to detect FliF, FliG, and RpoA, and 25 μl of WCLs was analyzed to detect FlrB. Proteins were detected with specific guinea pig antisera generated as described above. Primary antisera were applied to immunoblots for 1 to 2 h and used at the following concentrations: V. cholerae FliF UTGP151 (1:1,000), V. cholerae FliG UTGP198 (1:1,000), V. cholerae FlrB UTGP151 (1:2,000), V. cholerae RpoA UTGP197 (1:2,000), and P. aeruginosa FliG UTGP145 (1:1,000). A 1:10,000 dilution of horseradish peroxidase-conjugated goat anti-guinea pig antibody was then applied for detection of proteins.

### Electron microscopy analysis.

Overnight cultures of V. cholerae strains were inoculated into 5 ml LB at a 1:50 dilution and grown at 37°C with shaking to an OD_600_ of approximately 0.8. One milliliter of each culture was pelleted for 3 min at 13,200 rpm in a microcentrifuge, resuspended in 2% glutaraldehyde in 0.1 M cacodylate, and then incubated on ice for 1 h. Copper-coated Formvar grids were negatively glow discharged, and bacterial samples then were applied to the grids. The samples were stained with 2% uranyl acetate and visualized with an FEI Technai G2 Spirit Bio TWIN transmission electron microscope. Flagellum numbers were counted from at least 100 individual cells and averaged from three biological replicates to determine the proportion of bacterial populations producing different flagellation phenotypes: hyperflagellated (producing at two or more flagella at least at one pole), wild-type (producing a single flagellum at one pole), or aflagellated (lacking a flagellum). After averaging, the standard deviations for each population were calculated.

### Motility analysis.

V. cholerae strains were grown from freezer stocks in 5 ml LB overnight at 37°C with shaking. After growth, each strain was inoculated into LB motility agar (containing 0.3% agar) with an inoculation needle. Agar plates then were incubated for 8 h at 37°C.

### Statistical analysis.

Tests for significance in differences in expression of transcriptional reporter assays were conducted using the Student's *t* test (two-tailed distribution with two-sample, equal variance calculations). For analysis of flagellation of V. cholerae populations, a Student's *t* test (two-tailed distribution with two-sample, equal variance calculations) was used to evaluate statistical significance of monotrichous flagellation, aflagellation, or hyperflagellation between WT V. cholerae and transcriptional reprogramming mutants in the WT background and between the V. cholerae Δ*flhG* strains and transcriptional reprogramming mutants in the Δ*flhG* background. As indicated in the tables, figures, or figure legends, statistically significant differences between relevant strains possessed *P* values of <0.05.

### Data availability.

All data and methodologies are available upon request.

## References

[1] Kazmierczak BI, Hendrixson DR 2013 Spatial and numerical regulation of flagellar biosynthesis in polarly flagellated bacteria. Mol Microbiol 88:655–663. doi:10.1111/mmi.12221. 23600726 PMC3654036

[2] Schuhmacher JS, Thormann KM, Bange G 2015 How bacteria maintain location and number of flagella. FEMS Microbiol Rev 39:812–822. doi:10.1093/femsre/fuv034. 26195616

[3] Gulbronson CJ, Ribardo DA, Balaban M, Knauer C, Bange G, Hendrixson DR 2016 FlhG employs diverse intrinsic domains and influences FlhF GTPase activity to numerically regulate polar flagellar biogenesis in Campylobacter jejuni. Mol Microbiol 99:291–306. doi:10.1111/mmi.13231. 26411371 PMC4821507

[4] Ono H, Takashima A, Hirata H, Homma M, Kojima S 2015 The MinD homolog FlhG regulates the synthesis of the single polar flagellum of Vibrio alginolyticus. Mol Microbiol 98:130–141. doi:10.1111/mmi.13109. 26112286

[5] Kondo S, Imura Y, Mizuno A, Homma M, Kojima S 2018 Biochemical analysis of GTPase FlhF which controls the number and position of flagellar formation in marine Vibrio. Sci Rep 8:12115. doi:10.1038/s41598-018-30531-5. 30108243 PMC6092412

[6] Bange G, Kummerer N, Grudnik P, Lindner R, Petzold G, Kressler D, Hurt E, Wild K, Sinning I 2011 Structural basis for the molecular evolution of SRP-GTPase activation by protein. Nat Struct Mol Biol 18:1376–1380. doi:10.1038/nsmb.2141. 22056770

[7] Correa NE, Peng F, Klose KE 2005 Roles of the regulatory proteins FlhF and FlhG in the Vibrio cholerae flagellar transcription hierarchy. J Bacteriol 187:6324–6332. doi:10.1128/JB.187.18.6324-6332.2005. 16159765 PMC1236648

[8] Dasgupta N, Arora SK, Ramphal R 2000 fleN, a gene that regulates flagellar number in Pseudomonas aeruginosa. J Bacteriol 182:357–364. doi:10.1128/jb.182.2.357-364.2000. 10629180 PMC94283

[9] Dasgupta N, Ramphal R 2001 Interaction of the antiactivator FleN with the transcriptional activator FleQ regulates flagellar number in Pseudomonas aeruginosa. J Bacteriol 183:6636–6644. doi:10.1128/JB.183.22.6636-6644.2001. 11673434 PMC95495

[10] Kutsukake K, Ohya Y, Iino T 1990 Transcriptional analysis of the flagellar regulon of Salmonella typhimurium. J Bacteriol 172:741–747. doi:10.1128/jb.172.2.741-747.1990. 2404955 PMC208501

[11] Karlinsey JE, Tanaka S, Bettenworth V, Yamaguchi S, Boos W, Aizawa S-I, Hughes KT 2000 Completion of the hook-basal body complex of the Salmonella typhimurium flagellum is coupled to FlgM secretion and fliC transcription. Mol Microbiol 37:1220–1231. doi:10.1046/j.1365-2958.2000.02081.x. 10972838

[12] Chevance FF, Hughes KT 2008 Coordinating assembly of a bacterial macromolecular machine. Nat Rev Microbiol 6:455–465. doi:10.1038/nrmicro1887. 18483484 PMC5963726

[13] Hughes KT, Gillen KL, Semon MJ, Karlinsey JE 1993 Sensing structural intermediates in bacterial flagellar assembly by export of a negative regulator. Science 262:1277–1280. doi:10.1126/science.8235660. 8235660

[14] Kutsukake K 1997 Autogenous and global control of the flagellar master operon, flhD, in Salmonella typhimurium. Mol Gen Genet 254:440–448. doi:10.1007/s004380050437. 9180698

[15] Liu X, Matsumura P 1994 The FlhD/FlhC complex, a transcriptional activator of the Escherichia coli flagellar class II operons. J Bacteriol 176:7345–7351. doi:10.1128/jb.176.23.7345-7351.1994. 7961507 PMC197124

[16] Wang S, Fleming RT, Westbrook EM, Matsumura P, McKay DB 2006 Structure of the Escherichia coli FlhDC complex, a prokaryotic heteromeric regulator of transcription. J Mol Biol 355:798–808. doi:10.1016/j.jmb.2005.11.020. 16337229

[17] Klose KE, Mekalanos JJ 1998 Distinct roles of an alternative sigma factor during both free-swimming and colonizing phases of the Vibrio cholerae pathogenic cycle. Mol Microbiol 28:501–520. doi:10.1046/j.1365-2958.1998.00809.x. 9632254

[18] Prouty MG, Correa NE, Klose KE 2001 The novel σ^54^- and σ^28^-dependent flagellar gene transcription hierarchy of Vibrio cholerae. Mol Microbiol 39:1595–1609. doi:10.1046/j.1365-2958.2001.02348.x. 11260476

[19] Dasgupta N, Wolfgang MC, Goodman AL, Arora SK, Jyot J, Lory S, Ramphal R 2003 A four-tiered transcriptional regulatory circuit controls flagellar biogenesis in Pseudomonas aeruginosa. Mol Microbiol 50:809–824. doi:10.1046/j.1365-2958.2003.03740.x. 14617143

[20] Arora SK, Ritchings BW, Almira EC, Lory S, Ramphal R 1997 A transcriptional activator, FleQ, regulates mucin adhesion and flagellar gene expression in Pseudomonas aeruginosa in a cascade manner. J Bacteriol 179:5574–5581. doi:10.1128/jb.179.17.5574-5581.1997. 9287015 PMC179431

[21] Minamino T, Macnab RM 1999 Components of the Salmonella flagellar export apparatus and classification of export substrates. J Bacteriol 181:1388–1394. doi:10.1128/JB.181.5.1388-1394.1999. 10049367 PMC93525

[22] Li H, Sourjik V 2011 Assembly and stability of flagellar motor in Escherichia coli. Mol Microbiol 80:886–899. doi:10.1111/j.1365-2958.2011.07557.x. 21244534

[23] Morimoto YV, Ito M, Hiraoka KD, Che YS, Bai F, Kami-Ike N, Namba K, Minamino T 2014 Assembly and stoichiometry of FliF and FlhA in Salmonella flagellar basal body. Mol Microbiol 91:1214–1226. doi:10.1111/mmi.12529. 24450479

[24] Fukumura T, Makino F, Dietsche T, Kinoshita M, Kato T, Wagner S, Namba K, Imada K, Minamino T 2017 Assembly and stoichiometry of the core structure of the bacterial flagellar type III export gate complex. PLoS Biol 15:e2002281. doi:10.1371/journal.pbio.2002281. 28771466 PMC5542437

[25] Fabiani FD, Renault TT, Peters B, Dietsche T, Galvez EJC, Guse A, Freier K, Charpentier E, Strowig T, Franz-Wachtel M, Macek B, Wagner S, Hensel M, Erhardt M 2017 A flagellum-specific chaperone facilitates assembly of the core type III export apparatus of the bacterial flagellum. PLoS Biol 15:e2002267. doi:10.1371/journal.pbio.2002267. 28771474 PMC5542435

[26] Ohnishi K, Kutsukake K, Suzuki H, Iino T 1990 Gene fliA encodes an alternative sigma factor specific for flagellar operons in Salmonella typhimurium. Mol Gen Genet 221:139–147. doi:10.1007/bf00261713. 2196428

[27] Ohnishi K, Kutsukake K, Suzuki H, Lino T 1992 A novel transcriptional regulation mechanism in the flagellar regulon of Salmonella typhimurium: an anti-sigma factor inhibits the activity of the flagellum-specific sigma factor, σ^F^. Mol Microbiol 6:3149–3157. doi:10.1111/j.1365-2958.1992.tb01771.x. 1453955

[28] Chadsey MS, Karlinsey JE, Hughes KT 1998 The flagellar anti-σ factor FlgM actively dissociates Salmonella typhimurium σ^28^ RNA polymerase holoenzyme. Genes Dev 12:3123–3136. doi:10.1101/gad.12.19.3123. 9765212 PMC317189

[29] Chadsey MS, Hughes KT 2001 A multipartite interaction between Salmonella transcription factor sigma28 and its anti-sigma factor FlgM: implications for sigma28 holoenzyme destabilization through stepwise binding. J Mol Biol 306:915–929. doi:10.1006/jmbi.2001.4438. 11237608

[30] Kutsukake K 1994 Excretion of the anti-sigma factor through a flagellar substructure couples flagellar gene expression with flagellar assembly in Salmonella typhimurium. Mol Gen Genet 243:605–612. doi:10.1007/bf00279569. 8028576

[31] Hendrixson DR, DiRita VJ 2003 Transcription of σ^54^-dependent but not σ^28^-dependent flagellar genes in Campylobacter jejuni is associated with formation of the flagellar secretory apparatus. Mol Microbiol 50:687–702. doi:10.1046/j.1365-2958.2003.03731.x. 14617189

[32] Boll JM, Hendrixson DR 2013 A regulatory checkpoint during flagellar biogenesis in Campylobacter jejuni initiates signal transduction to activate transcription of flagellar genes. mBio 4:e00432. doi:10.1128/mBio.00432-13. 24003178 PMC3760246

[33] Joslin SN, Hendrixson DR 2009 Activation of the Campylobacter jejuni FlgSR two-component system is linked to the flagellar export apparatus. J Bacteriol 191:2656–2667. doi:10.1128/JB.01689-08. 19201799 PMC2668382

[34] Spohn G, Scarlato V 1999 Motility of Helicobacter pylori is coordinately regulated by the transcriptional activator FlgR, an NtrC homolog. J Bacteriol 181:593–599. doi:10.1128/JB.181.2.593-599.1999. 9882675 PMC93415

[35] Brahmachary P, Dashti MG, Olson JW, Hoover TR 2004 Helicobacter pylori FlgR is an enhancer-independent activator of σ^54^-RNA polymerase holoenzyme. J Bacteriol 186:4535–4542. doi:10.1128/JB.186.14.4535-4542.2004. 15231786 PMC438555

[36] Niehus E, Gressmann H, Ye F, Schlapbach R, Dehio M, Dehio C, Stack A, Meyer TF, Suerbaum S, Josenhans C 2004 Genome-wide analysis of transcriptional hierarchy and feedback regulation in the flagellar system of Helicobacter pylori. Mol Microbiol 52:947–961. doi:10.1111/j.1365-2958.2004.04006.x. 15130117

[37] Beier D, Frank R 2000 Molecular characterization of two-component systems of Helicobacter pylori. J Bacteriol 182:2068–2076. doi:10.1128/jb.182.8.2068-2076.2000. 10735847 PMC111253

[38] Schmitz A, Josenhans C, Suerbaum S 1997 Cloning and characterization of the Helicobacter pylori flbA gene, which codes for a membrane protein involved in coordinated expression of flagellar genes. J Bacteriol 179:987–997. doi:10.1128/jb.179.4.987-997.1997. 9023175 PMC178789

[39] Smith TG, Pereira L, Hoover TR 2009 Helicobacter pylori FlhB processing-deficient variants affect flagellar assembly but not flagellar gene expression. Microbiology 155:1170–1180. doi:10.1099/mic.0.022806-0. 19332819

[40] Tsang J, Hirano T, Hoover TR, McMurry JL 2015 Helicobacter pylori FlhA binds the sensor kinase and flagellar gene regulatory protein FlgS with high affinity. J Bacteriol 197:1886–1892. doi:10.1128/JB.02610-14. 25802298 PMC4420913

[41] Tsang J, Hoover TR 2015 Basal body structures differentially affect transcription of RpoN- and FliA-dependent flagellar genes in Helicobacter pylori. J Bacteriol 197:1921–1930. doi:10.1128/JB.02533-14. 25825427 PMC4420910

[42] Tsang J, Hoover TR 2014 Requirement of the flagellar protein export apparatus component FliO for optimal expression of flagellar genes in Helicobacter pylori. J Bacteriol 196:2709–2717. doi:10.1128/JB.01332-13. 24837287 PMC4135663

[43] Tsang J, Smith TG, Pereira LE, Hoover TR 2013 Insertion mutations in Helicobacter pylori flhA reveal strain differences in RpoN-dependent gene expression. Microbiology 159:58–67. doi:10.1099/mic.0.059063-0. 23154969 PMC3542725

[44] Correa NE, Lauriano CM, McGee R, Klose KE 2000 Phosphorylation of the flagellar regulatory protein FlrC is necessary for Vibrio cholerae motility and enhanced colonization. Mol Microbiol 35:743–755. doi:10.1046/j.1365-2958.2000.01745.x. 10692152

[45] Correa NE, Klose KE 2005 Characterization of enhancer binding by the Vibrio cholerae flagellar regulatory protein FlrC. J Bacteriol 187:3158–3170. doi:10.1128/JB.187.9.3158-3170.2005. 15838043 PMC1082837

[46] Joslin SN, Hendrixson DR 2008 Analysis of the Campylobacter jejuni FlgR response regulator suggests integration of diverse mechanisms to activate an NtrC-like protein. J Bacteriol 190:2422–2433. doi:10.1128/JB.01827-07. 18223079 PMC2293185

[47] Boll JM, Hendrixson DR 2011 A specificity determinant for phosphorylation in a response regulator prevents in vivo cross-talk and modification by acetyl phosphate. Proc Natl Acad Sci U S A 108:20160–20165. doi:10.1073/pnas.1113013108. 22128335 PMC3250149

[48] Pruss BM, Liu X, Hendrickson W, Matsumura P 2001 FlhD/FlhC-regulated promoters analyzed by gene array and lacZ gene fusions. FEMS Microbiol Lett 197:91–97. doi:10.1111/j.1574-6968.2001.tb10588.x. 11287152

[49] Homma M, Komeda Y, Iino T, Macnab RM 1987 The flaFIX gene product of Salmonella typhimurium is a flagellar basal body component with a signal peptide for export. J Bacteriol 169:1493–1498. doi:10.1128/jb.169.4.1493-1498.1987. 3549691 PMC211974

[50] Jones CJ, Homma M, Macnab RM 1989 L-, P-, and M-ring proteins of the flagellar basal body of Salmonella typhimurium: gene sequences and deduced protein sequences. J Bacteriol 171:3890–3900. doi:10.1128/jb.171.7.3890-3900.1989. 2544561 PMC210140

[51] Cohen EJ, Hughes KT 2014 Rod-to-hook transition for extracellular flagellum assembly is catalyzed by the L-ring-dependent rod scaffold removal. J Bacteriol 196:2387–2395. doi:10.1128/JB.01580-14. 24748615 PMC4054162

[52] Richter GW, Kress Y 1967 Electron microscopy of a strain of Bordetella bronchiseptica. J Bacteriol 94:1216–1224. doi:10.1128/JB.94.4.1216-1224.1967. 4167588 PMC276796

[53] Nierman WC, DeShazer D, Kim HS, Tettelin H, Nelson KE, Feldblyum T, Ulrich RL, Ronning CM, Brinkac LM, Daugherty SC, Davidsen TD, Deboy RT, Dimitrov G, Dodson RJ, Durkin AS, Gwinn ML, Haft DH, Khouri H, Kolonay JF, Madupu R, Mohammoud Y, Nelson WC, Radune D, Romero CM, Sarria S, Selengut J, Shamblin C, Sullivan SA, White O, Yu Y, Zafar N, Zhou L, Fraser CM 2004 Structural flexibility in the Burkholderia mallei genome. Proc Natl Acad Sci U S A 101:14246–14251. doi:10.1073/pnas.0403306101. 15377793 PMC521142

[54] Hu RM, Yang TC, Yang SH, Tseng YH 2005 Deduction of upstream sequences of Xanthomonas campestris flagellar genes responding to transcription activation by FleQ. Biochem Biophys Res Commun 335:1035–1043. doi:10.1016/j.bbrc.2005.07.171. 16111660

[55] Chovatia M, Sikorski J, Schroder M, Lapidus A, Nolan M, Tice H, Glavina Del Rio T, Copeland A, Cheng JF, Lucas S, Chen F, Bruce D, Goodwin L, Pitluck S, Ivanova N, Mavromatis K, Ovchinnikova G, Pati A, Chen A, Palaniappan K, Land M, Hauser L, Chang YJ, Jeffries CD, Chain P, Saunders E, Detter JC, Brettin T, Rohde M, Goker M, Spring S, Bristow J, Markowitz V, Hugenholtz P, Kyrpides NC, Klenk HP, Eisen JA 2009 Complete genome sequence of Thermanaerovibrio acidaminovorans type strain (Su883). Stand Genomic Sci 1:254–261. doi:10.4056/sigs.40645. 21304665 PMC3035242

[56] Baker MD, Wolanin PM, Stock JB 2006 Signal transduction in bacterial chemotaxis. Bioessays 28:9–22. doi:10.1002/bies.20343. 16369945

[57] Gosink KK, Kobayashi R, Kawagishi I, Hase CC 2002 Analyses of the roles of the three cheA homologs in chemotaxis of Vibrio cholerae. J Bacteriol 184:1767–1771. doi:10.1128/jb.184.6.1767-1771.2002. 11872729 PMC134905

[58] Zschiedrich CP, Keidel V, Szurmant H 2016 Molecular mechanisms of two-component signal transduction. J Mol Biol 428:3752–3775. doi:10.1016/j.jmb.2016.08.003. 27519796 PMC5023499

[59] Krell T, Lacal J, Busch A, Silva-Jimenez H, Guazzaroni ME, Ramos JL 2010 Bacterial sensor kinases: diversity in the recognition of environmental signals. Annu Rev Microbiol 64:539–559. doi:10.1146/annurev.micro.112408.134054. 20825354

[60] Klose KE, Mekalanos JJ 1998 Differential regulation of multiple flagellins in Vibrio cholerae. J Bacteriol 180:303–316. doi:10.1128/JB.180.2.303-316.1998. 9440520 PMC106886

[61] Kihara M, Minamino T, Yamaguchi S, Macnab RM 2001 Intergenic suppression between the flagellar MS ring protein FliF of Salmonella and FlhA, a membrane component of its export apparatus. J Bacteriol 183:1655–1662. doi:10.1128/JB.183.5.1655-1662.2001. 11160096 PMC95050

[62] Kihara M, Miller GU, Macnab RM 2000 Deletion analysis of the flagellar switch protein FliG of Salmonella. J Bacteriol 182:3022–3028. doi:10.1128/jb.182.11.3022-3028.2000. 10809678 PMC94485

[63] Paul K, Gonzalez-Bonet G, Bilwes AM, Crane BR, Blair D 2011 Architecture of the flagellar rotor. EMBO J 30:2962–2971. doi:10.1038/emboj.2011.188. 21673656 PMC3160249

[64] Lee LK, Ginsburg MA, Crovace C, Donohoe M, Stock D 2010 Structure of the torque ring of the flagellar motor and the molecular basis for rotational switching. Nature 466:996–1000. doi:10.1038/nature09300. 20676082 PMC3159035

[65] Ardissone S, Viollier PH 2015 Interplay between flagellation and cell cycle control in Caulobacter. Curr Opin Microbiol 28:83–92. doi:10.1016/j.mib.2015.08.012. 26476805

[66] Quon KC, Marczynski GT, Shapiro L 1996 Cell cycle control by an essential bacterial two-component signal transduction protein. Cell 84:83–93. doi:10.1016/s0092-8674(00)80995-2. 8548829

[67] Wingrove JA, Mangan EK, Gober JW 1993 Spatial and temporal phosphorylation of a transcriptional activator regulates pole-specific gene expression in Caulobacter. Genes Dev 7:1979–1992. doi:10.1101/gad.7.10.1979. 8406002

[68] Benson AK, Ramakrishnan G, Ohta N, Feng J, Ninfa AJ, Newton A 1994 The Caulobacter crescentus FlbD protein acts at ftr sequence elements both to activate and to repress transcription of cell cycle-regulated flagellar genes. Proc Natl Acad Sci U S A 91:4989–4993. doi:10.1073/pnas.91.11.4989. 8197169 PMC43915

[69] Newton A, Ohta N, Ramakrishnan G, Mullin D, Raymond G 1989 Genetic switching in the flagellar gene hierarchy of Caulobacter requires negative as well as positive regulation of transcription. Proc Natl Acad Sci U S A 86:6651–6655. doi:10.1073/pnas.86.17.6651. 2771949 PMC297903

[70] Ramakrishnan G, Newton A 1990 FlbD of Caulobacter crescentus is a homologue of the NtrC (NRI) protein and activates sigma 54-dependent flagellar gene promoters. Proc Natl Acad Sci U S A 87:2369–2373. doi:10.1073/pnas.87.6.2369. 2315326 PMC53688

[71] Mangan EK, Bartamian M, Gober JW 1995 A mutation that uncouples flagellum assembly from transcription alters the temporal pattern of flagellar gene expression in Caulobacter crescentus. J Bacteriol 177:3176–3184. doi:10.1128/jb.177.11.3176-3184.1995. 7768816 PMC177008

[72] Muir RE, Easter J, Gober JW 2005 The trans-acting flagellar regulatory proteins, FliX and FlbD, play a central role in linking flagellar biogenesis and cytokinesis in Caulobacter crescentus. Microbiology 151:3699–3711. doi:10.1099/mic.0.28174-0. 16272391

[73] Muir RE, Gober JW 2002 Mutations in FlbD that relieve the dependency on flagellum assembly alter the temporal and spatial pattern of developmental transcription in Caulobacter crescentus. Mol Microbiol 43:597–615. doi:10.1046/j.1365-2958.2002.02728.x. 11929518

[74] Muir RE, Gober JW 2004 Regulation of FlbD activity by flagellum assembly is accomplished through direct interaction with the trans-acting factor, FliX. Mol Microbiol 54:715–730. doi:10.1111/j.1365-2958.2004.04298.x. 15491362

[75] Muir RE, O'Brien TM, Gober JW 2001 The Caulobacter crescentus flagellar gene, fliX, encodes a novel trans-acting factor that couples flagellar assembly to transcription. Mol Microbiol 39:1623–1637. doi:10.1046/j.1365-2958.2001.02351.x. 11260478

[76] Balaban M, Hendrixson DR 2011 Polar flagellar biosynthesis and a regulator of flagellar number influence spatial parameters of cell division in Campylobacter jejuni. PLoS Pathog 7:e1002420. doi:10.1371/journal.ppat.1002420. 22144902 PMC3228812

[77] Huitema E, Pritchard S, Matteson D, Radhakrishnan SK, Viollier PH 2006 Bacterial birth scar proteins mark future flagellum assembly site. Cell 124:1025–1037. doi:10.1016/j.cell.2006.01.019. 16530048

[78] Obuchowski PL, Jacobs-Wagner C 2008 PflI, a protein involved in flagellar positioning in Caulobacter crescentus. J Bacteriol 190:1718–1729. doi:10.1128/JB.01706-07. 18165296 PMC2258662

[79] Lam H, Schofield WB, Jacobs-Wagner C 2006 A landmark protein essential for establishing and perpetuating the polarity of a bacterial cell. Cell 124:1011–1023. doi:10.1016/j.cell.2005.12.040. 16530047

[80] Davis NJ, Cohen Y, Sanselicio S, Fumeaux C, Ozaki S, Luciano J, Guerrero-Ferreira RC, Wright ER, Jenal U, Viollier PH 2013 De- and repolarization mechanism of flagellar morphogenesis during a bacterial cell cycle. Genes Dev 27:2049–2062. doi:10.1101/gad.222679.113. 24065770 PMC3792480

[81] Schuhmacher JS, Rossmann F, Dempwolff F, Knauer C, Altegoer F, Steinchen W, Dorrich AK, Klingl A, Stephan M, Linne U, Thormann KM, Bange G 2015 MinD-like ATPase FlhG effects location and number of bacterial flagella during C-ring assembly. Proc Natl Acad Sci U S A 112:3092–3097. doi:10.1073/pnas.1419388112. 25733861 PMC4364217

[82] Green JC, Kahramanoglou C, Rahman A, Pender AM, Charbonnel N, Fraser GM 2009 Recruitment of the earliest component of the bacterial flagellum to the old cell division pole by a membrane-associated signal recognition particle family GTP-binding protein. J Mol Biol 391:679–690. doi:10.1016/j.jmb.2009.05.075. 19497327

[83] Leipe DD, Wolf YI, Koonin EV, Aravind L 2002 Classification and evolution of P-loop GTPases and related ATPases. J Mol Biol 317:41–72. doi:10.1006/jmbi.2001.5378. 11916378

[84] Bange G, Sinning I 2013 SIMIBI twins in protein targeting and localization. Nat Struct Mol Biol 20:776–780. doi:10.1038/nsmb.2605. 23823071

[85] Bange G, Petzold G, Wild K, Parlitz RO, Sinning I 2007 The crystal structure of the third signal-recognition particle GTPase FlhF reveals a homodimer with bound GTP. Proc Natl Acad Sci U S A 104:13621–13625. doi:10.1073/pnas.0702570104. 17699634 PMC1959431

[86] Zheng J, Shin OS, Cameron DE, Mekalanos JJ 2010 Quorum sensing and a global regulator TsrA control expression of type VI secretion and virulence in Vibrio cholerae. Proc Natl Acad Sci U S A 107:21128–21133. doi:10.1073/pnas.1014998107. 21084635 PMC3000250

[87] Rahme LG, Stevens EJ, Wolfort SF, Shao J, Tompkins RG, Ausubel FM 1995 Common virulence factors for bacterial pathogenicity in plants and animals. Science 268:1899–1902. doi:10.1126/science.7604262. 7604262

[88] Lee DG, Urbach JM, Wu G, Liberati NT, Feinbaum RL, Miyata S, Diggins LT, He J, Saucier M, Deziel E, Friedman L, Li L, Grills G, Montgomery K, Kucherlapati R, Rahme LG, Ausubel FM 2006 Genomic analysis reveals that Pseudomonas aeruginosa virulence is combinatorial. Genome Biol 7:R90. doi:10.1186/gb-2006-7-10-r90. 17038190 PMC1794565

[89] Henderson MJ, Milazzo FH 1979 Arylsulfatase in Salmonella typhimurium: detection and influence of carbon source and tyramine on its synthesis. J Bacteriol 139:80–87. doi:10.1128/JB.139.1.80-87.1979. 222733 PMC216829

[90] Yao R, Guerry P 1996 Molecular cloning and site-specific mutagenesis of a gene involved in arylsulfatase production in Campylobacter jejuni. J Bacteriol 178:3335–3338. doi:10.1128/jb.178.11.3335-3338.1996. 8655516 PMC178088

[91] Miller J 1972 Experiments in molecular genetics. Cold Spring Harbor Laboratory, Cold Spring Harbor, NY.

[92] Barrero-Tobon AM, Hendrixson DR 2012 Identification and analysis of flagellar coexpressed determinants (Feds) of Campylobacter jejuni involved in colonization. Mol Microbiol 84:352–369. doi:10.1111/j.1365-2958.2012.08027.x. 22375824 PMC3323700

[93] Barrero-Tobon AM, Hendrixson DR 2014 Flagellar biosynthesis exerts temporal regulation of secretion of specific Campylobacter jejuni colonization and virulence determinants. Mol Microbiol 93:957–974. doi:10.1111/mmi.12711. 25041103 PMC4150830

