# Supplementary figures and images for: A Polar Flagellar Transcriptional Program Mediated by Diverse Two-Component Signal Transduction Systems and Basal Flagellar Proteins Is Broadly Conserved in Polar Flagellates
Source: mBio. 2020 Mar 3;11(2):e03107-19. doi: 10.1128/mBio.03107-19 (PMC7064773; doi:10.1128/mBio.03107-19)

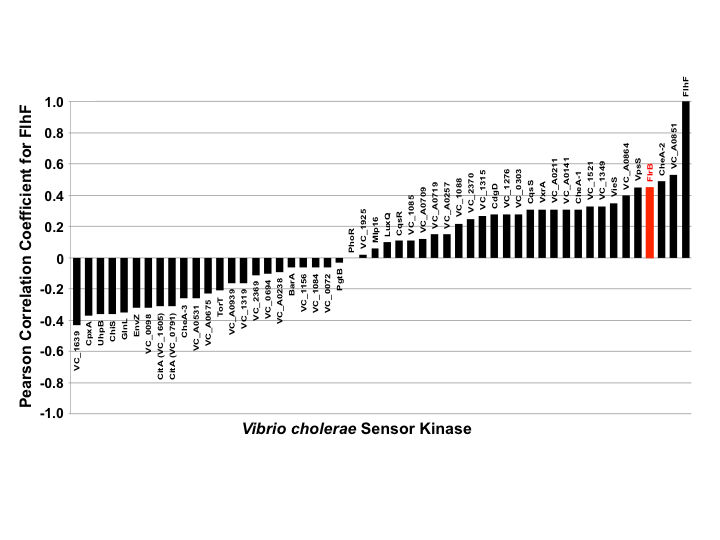

Supplement: FIG S1 [file mBio.03107-19-sf001.tif]

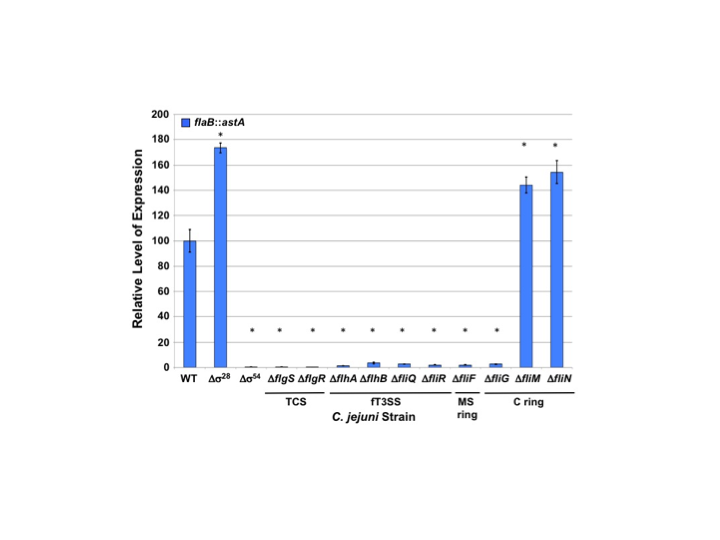

Supplement: FIG S2 [file mBio.03107-19-sf002.tif]

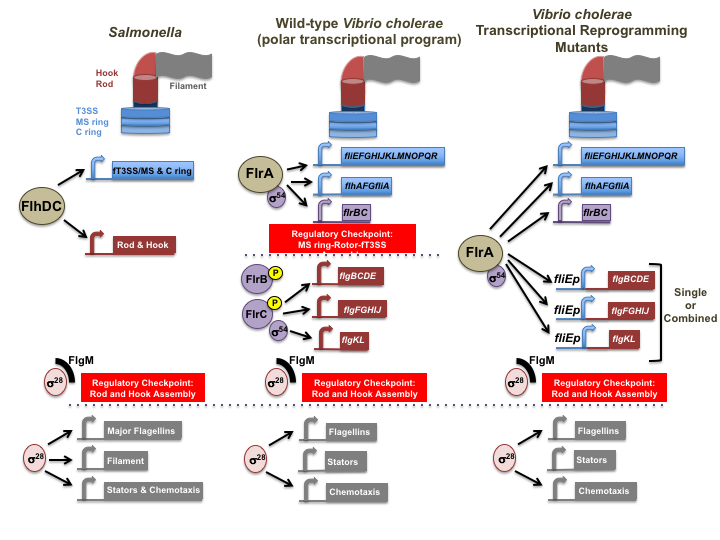

Supplement: FIG S3 [file mBio.03107-19-sf003.tif]
