# Supplementary material for: A Polar Flagellar Transcriptional Program Mediated by Diverse Two-Component Signal Transduction Systems and Basal Flagellar Proteins Is Broadly Conserved in Polar Flagellates
Source: mBio. 2020 Mar 3;11(2):e03107-19. doi: 10.1128/mBio.03107-19 (PMC7064773; doi:10.1128/mBio.03107-19)
Supplement: TEXT S1 [file mBio.03107-19-s0001.pdf]

Text S1. Additional Materials and Methods

Bacterial strain and plasmid construction. All bacterial strains and plasmids used in this study are listed in Tables S1 and S2. All plasmids for mutagenesis or harboring transcriptional reporters were sequenced after construction to verify correct design of plasmids.

C. jejuni. Creation of *C. jejuni* 81-176 *rpsL*^{Sm} Δ*astA* Δ*fliM flaB*::*astA* (PMB979) followed previously described procedures to delete *astA* from the chromosome of 81-176 *rpsL*^{Sm} Δ*fliM* (CRG1005; (1)) with a two-step electroporation and selection procedure (2). After recovery of DRH6747 (81-176 *rpsL*^{Sm} Δ*astA* Δ*fliM*), the strain was electroporated with pDRH665 to replace *flaB* with *flaB*::*astA-kan* on the chromosome. Transformants were recovered on kanamycin and screened by colony PCR to result in PMB979.

V. cholerae. *V. cholerae* C6706 *lacZ* isogenic mutants used in this study were derivatives of specific TnFGL3 mutants from a defined transposon mutant library or generated by deletion of specific genes from the C6706 *lacZ* chromosome (3).

V. cholerae flrA, *flrB*, *flrC*, *fliF*, *flhB*, *fliP, fliQ*, *fliR*, *fliA*, and *rpoN* mutants were derived from mutants containing a TnFGL3 insertion (which encodes kanamycin-resistance) in each gene. Each TnFGL3 mutant was prepared for electroporation. Briefly, 100 ml LB containing kanamycin were inoculated with a 1:40 dilution of overnight cultures of each mutant and grown with shaking at 37°C to OD600 0.8. Bacteria were recovered by centrifugation, washed twice in cold 2 mM $CaCl_2$, washed once in 2 mM $CaCl_2$ with 10% glycerol, and then resuspended in 300 µl 2 mM $CaCl_2$ with 10% glycerol. Approximately 0.4 µg of pFlpE was electroporated into each mutant and the bacteria were grown in LB for 1 h at 37°C before plating on LB with ampicillin for selection. Ampicillin-resistant colonies were grown overnight at 37°C in 10 ml LB with 0.1% arabinose to induce FLP-mediated recombination to remove a large part of TnFGL3, including

the kanamycin-resistance gene, leaving a 192-bp scar within each gene to disrupt the coding sequence. Ten-fold serial dilutions were plated on LB with streptomycin and 40 μg/ml X-gal (5-bromo-4-chloro-3-indolyl-D-galactopyranoside). White, streptomycin-resistant colonies were then screened for sensitivity to ampicillin and kanamycin and then screened by colony PCR to verify the presence of the 192-bp scar within the coding sequence of each gene.

In-frame deletion of *flhA*, *fliQ*, *fliM*, *fliN*, or *fliG* from the chromosome of C6706 *lacZ* was accomplished by first creating pKAS32-based plasmids that contained DNA for the correct in-frame mutation (Table S2). Primers with 5' sites for restriction enzymes were designed to amplify from the C6706 *lacZ* genome two DNA fragments with 700-1000 nucleotides upstream and downstream of the portion of the gene to be deleted. These fragments were joined together by a second round of PCR and then cloned as a single fragment into the pKAS32 with T4 DNA ligase (New England Biolabs). DNA fragments for in-frame deletion of *flhG* or deletion of domains encoded by *fliG* were generated similarly by PCR and ligated into pGP704sacB28 with Gibson Assembly Mastermix (New England Biolabs).

To delete genes from the chromosome of C6706 *lacZ* with pKAS32-based plasmids, the respective plasmids were transformed into *E. coli* SM10 λ*pir*. C6706 *lacZ* and SM10 λ*pir* strains were inoculated from overnight cultures into LB at a 1:20 dilution and grown at 37°C with shaking to OD600 0.6 - 0.8. C6706 *lacZ* was mixed with SM10 λ*pir* strains at a ratio of 20:1 and collected onto a filter by vacuum using a manifold. The filters were placed on LB agar for 7 h at 37°C. Bacteria were collected from filters by washing with 2.5 ml PBS and 100 μl was plated on TCBS agar with ampicillin to select for C6706 *lacZ* transconjugants. After overnight incubation at 37°C for 24 h, transconjugants were streaked for individual colonies on LB with ampicillin. Colonies were screened for ampicillin resistance and streptomycin sensitivity and then grown

overnight in LB without antibiotics. Ten-fold dilutions of overnight cultures were plated on LB with 1 mg/ml streptomycin. Colonies were screened for ampicillin resistance and by colony PCR to verify generation of the correct in-frame deletion of the specific gene.

For deletion of *flhG* or portions of *fliG* from the chromosome of C6706 *lacZ*, pGP704sacB28 derivatives containing mutations were first transformed into *E. coli* SM10 λ*pir*. C6706 *lacZ* and SM10 λ*pir* strains were grown overnight at 30°C in LB without NaCl with appropriate antibiotics. One ml of SM10 λ*pir* strains were collected by centrifugation and then resuspended with 1 ml of the C6706 *lacZ* culture. After collection of the bacteria by centrifugation, bacteria were resuspended in 150 µl LB and 50 µl was spotted onto LB agar without NaCl or antibiotics. After overnight incubation at 37°C, each spot was collected in 900 µl LB and vortexed. Ten-fold serial dilutions were plated on LB agar without NaCl, but containing ampicillin and then agar plates were incubated overnight at 30°C. A colony from each transconjugant was restreaked on the same agar. A colony was then grown overnight in LB without NaCl at 30°C. A loopful of culture was streaked on LB agar containing 10% sucrose and lacking NaCl. Agar plates were incubated at room temperature for 36 - 48 h. Colonies were screened for streptomycin resistance and ampicillin sensitivity and then screened by colony PCR for generation of the correct in-frame deletion of the specific gene.

Construction of plasmids for creating mutants to express σ^{54}- and FlrBC-dependent flagellar rod and hook operons from the FlrA-dependent promoter of the *fliE* operon involved a tripartite fusion of three DNA fragments by PCR followed by insertion into the XbaI site of pGP704sacB28. The three DNAs that were fused together in the 5' to 3' orientation included: a region upstream of the promoters for the *flgB*, *flgF*, or *flgK* operons (-998 to -195, -1005 to -158, and -817 to -163, respectively); bases -89 to -1 upstream of the *fliE* coding sequence that

contains the FlrA-dependent *fliE* promoter; and the coding sequence of *flgB*, *flgF*, or *flgK*. The plasmids were sequenced and then transformed into *E. coli* SM10 λ*pir* for conjugation into C6706 *lacZ* or C6706 *lacZ* Δ*flhG* to replace the promoter for a specific operon with the promoter for the *fliE* operon on the chromosome. Conjugation and mutant isolation procedures are described as above. The procedures were repeated with resultant strains to replace two or three promoters for the rod and hook proteins with the *fliE* promoter on the *V. cholerae* chromosome.

Portions of the promoter regions of *flrA*, *flrB*, *flaA*, *flgB*, *flgF*, *flgK*, *fliE*, and *cheV* operons were amplified by PCR with primers SalI or BamHI sites at the 5' ends. These plasmids were then ligated into the SalI and BamHI sites of pTL61T with T4 DNA ligase to create *lacZ* transcriptional fusions to the promoters of these operons (Table S2). These plasmids were first transformed into DH5α and then electroporated into WT C6706 *lacZ* or isogenic mutants by procedures described above and recovered on LB agar with ampicillin. Transformants were screened by colony PCR for retention of the *lacZ* transcriptional fusions *in trans* on the pTL61T derivatives.

Plasmids to complement C6706 *lacZ* mutants were generated from pACYC184. DNA containing approximately 20 nucleotides upstream and downstream of the coding sequence of each gene was amplified by PCR with primers encoding 5' restriction sites. These fragments were cloned into BamHI- and SalI-digested pACYC184 or EcoRV-digested pACYC184 by T4 DNA ligase or Gibson Assembly Mastermix to disrupt the tetracycline-resistance gene and then transformed into DH5α. Complemented genes were thus expressed from the promoter of the tetracycline-resistance gene. pGP704sacB28-based plasmids carrying $fliF_{\Delta AS200\text{-}201}$ and $fliF_{\Delta AS202\text{-}203}$ were created by amplifying two DNA fragments with approximately 1.5 kb

upstream of the two codons to be deleted from *fliF*. These fragments were then joined together by PCR and then cloned into pGP704sacB28 using Gibson Assembly Mastermix (New England Biolabs). These plasmids were then used as template to amplify DNA to clone into pACYC184 for complementation. pACYC184 was also digested with EcoRV and NruI to remove a portion of the tetracycline-resistance gene and religated to create a negative control vector for complementation. Transformants were selected on LB with chloramphenicol. After verification of interruption of the tetracycline-resistance cassette, the plasmids were then electroporated into C6706 *lacZ* strains by procedures described above. Transformants were recovered on LB agar with chloramphenicol.

P. aeruginosa. In-frame deletion of genes from the chromosome of PA14 was accomplished by creating pEX18Gm-based plasmids that contained DNA for the correct in-frame mutation (Table S2). Primers with 5' sites for restriction enzymes were designed to amplify from the PA14 genome DNA fragments with 700-1000 nucleotides upstream of the portion of the gene to be deleted. These fragments were cloned into pEX18Gm by Gibson Assembly Mastermix or joined together by a second round of PCR and then cloned as a single fragment into pEX18Gm with Gibson Assembly Mastermix or T4 DNA ligase (New England Biolabs).

DNA was introduced by electroporation into PA14 to delete genes from the chromosome based on previously described procedures (4, 5). Briefly, PA14 was grown as overnight cultures in 6 ml LB at 37°C. Bacteria were collected by centrifugation at room temperature and washed three times in room temperature 300 mM sucrose. After the last centrifugation step, bacteria were resuspended in 200 µl of 300 mM sucrose. For electroporation, 100 µl of PA14 resuspension and 300 ng of each pEX18Gm-based plasmid were mixed and electroporated. Bacteria were collected and then grown in 1 ml LB broth without NaCl for 4 h at 37°C. After

growth, 100 μl of culture were plated on VBEM with 100 μg/ml gentamicin and then incubated at 30°C for 48 h. Transformants were patched on VBEM with 100 μg/ml gentamicin and VBEM with 10% sucrose and grown for 24 h at 30°C. Gentamicin-resistant and sucrose-sensitive colonies were then grown overnight at 37°C in LB without salt. Ten-fold serial dilutions were plated on VBEM with 10% sucrose and grown at 30°C for up to 48 h. Colonies were patched on LB agar without salt but containing 10% sucrose and LB with 100 μg/ml gentamicin at 37°C to identify sucrose-resistant and gentamicin-sensitive colonies. These colonies were then screened by colony PCR to verify generation of the correct in-frame deletion from the chromosome of PA14.

Promoter regions of *flgB* and *fliA* operons were amplified by PCR with primers containing restriction sites at the 5' ends. The *flgB* promoter was ligated as a SalI-BamHI fragment into mini-CTX-*lacZ* and the *fliA* promoter was ligated as a PstI-BamHI fragment into mini-CTX-*lacZ* with T4 DNA ligase to create *lacZ* transcriptional fusions to the promoters of these operons (Table S2). These plasmids were transformed into DH5α and transformants were selected for on LB with 12.5 μg/ml tetracycline. Plasmids were then transformed into *E. coli* SY17.1 λ*pir*.

WT PA14 and PA14 mutants were then conjugated with SY17.1 λ*pir* containing mini-CTX-*lacZ* harboring *flgB*- or *fliA*-*lacZ* transcriptional fusions. LB broth with tetracycline was inoculated with a 1:100 dilution of overnight culture of SY17.1 λ*pir* strains and then grown at 37°C with shaking to an OD600 0.3 - 0.6. Five-hundred microliters of overnight cultures of PA14 strains were inoculated into 10 ml LB without antibiotics and incubated without shaking at 42°C during growth of the SY17.1 λ*pir* strains. SY17.1 λ*pir* and PA14 cultures were then combined (0.5 ml of each) and collected by centrifugation. Bacterial pellets were suspended in

40 µl of LB broth and spotted on LB without antibiotics. After overnight incubation at 30°C, the spots were scraped into 1 ml PBS and 10-fold serial dilutions were plated on VBEM containing 100 µg/ml tetracycline. Bacteria were then incubated at 30°C for 48 h. Colonies were patched onto VBEM with tetracycline and grown at 37°C overnight. Colonies were verified by colony PCR to ensure integration of the mini-CTX-*lacZ* DNA into the *att* site on the chromosome and then frozen. To remove the backbone of mini-CTX-*lacZ*, PA14 strains were then conjugated with SY17.1 λ*pir* containing pFLP2, which harbors a plasmid for expression of the FLP recombinase. Conjugation was performed as described above except conjugation mixtures were initially spotted on LB without NaCl and then transconjugants were selected by plating 10-fold serial dilutions on VBEM containing 200 µg/ml carbenicillin. After growth for 48 h at 30°C, colonies were patched on VBEM containing 200 µg/ml carbenicillin or 100 µg/ml tetracycline. Carbenicillin-resistant and tetracycline-sensitive colonies were then grown in LB without NaCl or antibiotics at 37°C. After overnight growth, 10-fold serial dilutions were plated on VBEM with 10% sucrose and incubated for 2 h at 30°C. Colonies were then patched on VBEM containing 10% sucrose, 200 µg/ml carbenicillin, or 100 µg/ml tetracycline. Sucrose-resistant, carbenicillin-sensitive, and tetracycline-sensitive colonies were then screened by colony PCR to ensure retention of the *lacZ* transcriptional fusions at the *att* site on the chromosome and removal of the mini-CTX backbone from the genome.

References

1. **Henderson LD, Matthews-Palmer TRS, Gulbronson CJ, Ribardo, DA, Beeby M, Hendrixson DR.** Diversification of *Campylobacter jejuni* flagellar C ring composition impacts structure, flagellar biology and cellular processes. mBio, *in press*.
2. **Hendrixson DR, DiRita VJ.** 2003. Transcription of σ^{54}-dependent but not σ^{28}-dependent flagellar genes in *Campylobacter jejuni* is associated with formation of the flagellar secretory apparatus. Mol Microbiol **50:**687-702.
3. **Cameron DE, Urbach JM, Mekalanos JJ.** 2008. A defined transposon mutant library and its use in identifying motility genes in *Vibrio cholerae*. Proc Natl Acad Sci U S A **105:**8736-8741.
4. **Choi KH, Schweizer HP.** 2006. mini-Tn7 insertion in bacteria with single *attTn7* sites: example *Pseudomonas aeruginosa*. Nat Protoc **1:**153-161.
5. **Choi KH, Kumar A, Schweizer HP.** 2006. A 10-min method for preparation of highly electrocompetent *Pseudomonas aeruginosa* cells: application for DNA fragment transfer between chromosomes and plasmid transformation. J Microbiol Methods **64:**391-397.
6. **Hanahan D.** 1983. Studies on transformation of *Escherichia coli* with plasmids. J Mol Biol **166:**557-580.
7. **Miller VL, Mekalanos JJ.** 1988. A novel suicide vector and its use in construction of insertion mutations: osmoregulation of outer membrane proteins and virulence determinants in *Vibrio cholerae* requires *toxR*. J Bacteriol **170:**2575-2583.
8. **de Lorenzo V, Timmis KN.** 1994. Analysis and construction of stable phenotypes in gram-negative bacteria with Tn5- and Tn10-derived minitransposons. Methods Enzymol **235:**386-405.

9. **Bullock WO, Femandex JM, Short JM.** 1987. XL1-Blue: a high efficiency plasmid transforming *recA Escherichia coli* strain with beta-galactosidase selection. Biotechniques **5:**376-378.

10. **Boll JM, Hendrixson DR.** 2013. A regulatory checkpoint during flagellar biogenesis in *Campylobacter jejuni* initiates signal transduction to activate transcription of flagellar genes. mBio **4:**e00432-13.

11. **Joslin SN, Hendrixson DR.** 2009. Activation of the *Campylobacter jejuni* FlgSR two-component system is linked to the flagellar export apparatus. J Bacteriol **191:**2656-2667.

12. **Zheng J, Shin OS, Cameron DE, Mekalanos JJ.** 2010. Quorum sensing and a global regulator TsrA control expression of type VI secretion and virulence in *Vibrio cholerae*. Proc Natl Acad Sci U S A **107:**21128-21133.

13. **Lee DG, Urbach JM, Wu G, Liberati NT, Feinbaum RL, Miyata S, Diggins LT, He J, Saucier M, Deziel E, Friedman L, Li L, Grills G, Montgomery K, Kucherlapati R, Rahme LG, Ausubel FM.** 2006. Genomic analysis reveals that *Pseudomonas aeruginosa* virulence is combinatorial. Genome Biol **7:**R90.

14. **Rahme LG, Stevens EJ, Wolfort SF, Shao J, Tompkins RG, Ausubel FM.** 1995. Common virulence factors for bacterial pathogenicity in plants and animals. Science **268:**1899-1902.

15. **Hendrickson EL, Plotnikova J, Mahajan-Miklos S, Rahme LG, Ausubel FM.** 2001. Differential roles of the *Pseudomonas aeruginosa* PA14 *rpoN* gene in pathogenicity in plants, nematodes, insects, and mice. J Bacteriol **183:**7126-7134.

16. **Linn T, St Pierre R.** 1990. Improved vector system for constructing transcriptional fusions that ensures independent translation of *lacZ*. J Bacteriol **172:**1077-1084.

17. **Chang AC, Cohen SN.** 1978. Construction and characterization of amplifiable multicopy DNA cloning vehicles derived from the P15A cryptic miniplasmid. J Bacteriol **134:**1141-1156.

18. **Rose RE.** 1988. The nucleotide sequence of pACYC184. Nucleic Acids Res **16:**355.

19. **Skorupski K, Taylor RK.** 1996. Positive selection vectors for allelic exchange. Gene **169:**47-52.

20. **Becher A, Schweizer HP.** 2000. Integration-proficient *Pseudomonas aeruginosa* vectors for isolation of single-copy chromosomal *lacZ* and *lux* gene fusions. Biotechniques **29:**948-950, 952.

21. **Hoang TT, Karkhoff-Schweizer RR, Kutchma AJ, Schweizer HP.** 1998. A broad-host-range Flp-FRT recombination system for site-specific excision of chromosomally-located DNA sequences: application for isolation of unmarked *Pseudomonas aeruginosa* mutants. Gene **212:**77-86.
