# Supplementary material for: A Polar Flagellar Transcriptional Program Mediated by Diverse Two-Component Signal Transduction Systems and Basal Flagellar Proteins Is Broadly Conserved in Polar Flagellates
Source: mBio. 2020 Mar 3;11(2):e03107-19. doi: 10.1128/mBio.03107-19 (PMC7064773; doi:10.1128/mBio.03107-19)
Supplement: TABLE S1 [file mBio.03107-19-st001.pdf]

Table S1. Bacterial strains used in this study

Strain	Genotype	Source/Reference
E. coli **stains**		
DH5α	*E. coli supE44* Δ*lacU169* (φ80l*acZ*DM15) *hsdR17 recA1 endA1 gyrA96 thi-1 relA1*	Invitrogen
DH5α λ*pir*	F- Δ(*lacZYA-argF*)U169 *recA1 endA1 hsdR17 supE44 thi-1 gyrA96 relA1* λ::*pir*	(6)
SM10 λ*pir*	*thi thr leu tonA lacY supE recA*::RP-4-Tc::Mu(λ*pir*)	(7)
SY17.1 λ*pir*	*E. coli* Tp^r $rpsL^{Sm}$ *recA thi pro* r_K^- m_K^+ RP4:2-Tc:MuKm Tn7 λ*pir*	(8)
BL21 (DE3)	*E. coli fhuA2 [lon] ompT gal (λ DE3) [dcm] ΔhsdS λ DE3 = λ sBamHIo ΔEcoRI-B int::(lacI::PlacUV5::T7 gene1) i21 Δnin5*	New England Biolabs
XL1-Blue	F'::Tn*10 proA*$^+$*B*$^+$*lacI*^{q} *Δ(lacZ)M15/recA1 endA1 gyrA96* (Nal^r) *thi hsdR17* (r_K^- m_K^+) *glnV44 relA1 lac*	(9)
Campylobacter jejuni **strains**		
DRH665	81-176 $rpsL^{Sm}$ Δ*astA flaB*::*astA*	(2)
DRH667	81-176 $rpsL^{Sm}$ Δ*astA* Δ*rpoN flaB*::*astA*	(2)
DRH842	81-176 $rpsL^{Sm}$ Δ*astA* Δ*flgR flaB*::*astA*	(2)
DRH939	81-176 $rpsL^{Sm}$ Δ*astA* Δ*flgS flaB*::*astA*	(2)
DRH1049	81-176 $rpsL^{Sm}$ Δ*astA* Δ*flhA flaB*::*astA*	(2)
DRH1074	81-176 $rpsL^{Sm}$ Δ*astA* Δ*fliA flaB*::*astA*	(2)
DRH1723	81-176 $rpsL^{Sm}$ Δ*astA* Δ*fliR flaB*::*astA*	(10)
DRH1830	81-176 $rpsL^{Sm}$ Δ*astA* Δ*flhB flaB*::*astA*	(11)
DRH2113	81-176 $rpsL^{Sm}$ Δ*astA* Δ*fliF flaB*::*astA*	(10)
DRH6131	81-176 $rpsL^{Sm}$ Δ*fliM astA*::*cat-rpsL*	This study
DRH6747	81-176 $rpsL^{Sm}$ Δ*astA* Δ*fliM*	This study
CRG1005	81-176 $rpsL^{Sm}$ Δ*fliM*	(1)
DAR152	81-176 $rpsL^{Sm}$ Δ*astA* Δ*fliQ flaB*::*astA*	(10)
JMB1258	81-176 $rpsL^{Sm}$ Δ*astA* Δ*fliG flaB*::*astA*	(10)
JMB1741	81-176 $rpsL^{Sm}$ Δ*astA* Δ*fliN flaB*::*astA*	(10)
PMB979	81-176 $rpsL^{Sm}$ Δ*astA* Δ*fliM flaB*::*astA*	This study
Vibrio cholerae **strains**		
C6706	O1 El Tor biotype; $rpsL^{Sm}$ *lacZ*	(12)
DRH3504	C6706 $rpsL^{Sm}$ *lacZ rpoN*::ΔTn	This study
DRH3507	C6706 $rpsL^{Sm}$ *lacZ fliA*::ΔTn	This study
DRH3510	C6706 $rpsL^{Sm}$ *lacZ flrA*::ΔTn	This study
DRH3513	C6706 $rpsL^{Sm}$ *lacZ flrB*::ΔTn	This study
DRH3516	C6706 $rpsL^{Sm}$ *lacZ flrC*::ΔTn	This study
DRH3519	C6706 $rpsL^{Sm}$ *lacZ fliF*::ΔTn	This study
DRH3552	C6706 $rpsL^{Sm}$ *lacZ flhB*::ΔTn	This study

DRH3555	C6706 *rpsL*^{Sm} *lacZ fliP*::ΔTn	This study
DRH3561	C6706 *rpsL*^{Sm} *lacZ fliR*::ΔTn	This study
DRH3611	C6706 *rpsL*^{Sm} *lacZ*/pDRH3470	This study
DRH3615	C6706 *rpsL*^{Sm} *lacZ*/pDRH3471	This study
DRH3617	C6706 *rpsL*^{Sm} *lacZ*/pDRH3472	This study
DRH3635	C6706 *rpsL*^{Sm} *lacZ flrC*::ΔTn/pDRH3472	This study
DRH3648	C6706 *rpsL*^{Sm} *lacZ rpoN*::ΔTn/pDRH3470	This study
DRH3651	C6706 *rpsL*^{Sm} *lacZ rpoN*::ΔTn/pDRH3471	This study
DRH3654	C6706 *rpsL*^{Sm} *lacZ rpoN*::ΔTn/pDRH3472	This study
DRH3659	C6706 *rpsL*^{Sm} *lacZ fliA*::ΔTn/pDRH3470	This study
DRH3662	C6706 *rpsL*^{Sm} *lacZ fliA*::ΔTn/pDRH3471	This study
DRH3666	C6706 *rpsL*^{Sm} *lacZ fliA*::ΔTn/pDRH3472	This study
DRH3704	C6706 *rpsL*^{Sm} *lacZ flrA*::ΔTn/pDRH3470	This study
DRH3708	C6706 *rpsL*^{Sm} *lacZ flrA*::ΔTn/pDRH3471	This study
DRH3717	C6706 *rpsL*^{Sm} *lacZ flrB*::ΔTn/pDRH3470	This study
DRH3719	C6706 *rpsL*^{Sm} *lacZ flrA*::ΔTn/pDRH3472	This study
DRH3721	C6706 *rpsL*^{Sm} *lacZ flrB*::ΔTn/pDRH3471	This study
DRH3723	C6706 *rpsL*^{Sm} *lacZ flrB*::ΔTn/pDRH3472	This study
DRH3729	C6706 *rpsL*^{Sm} *lacZ flrC*::ΔTn/pDRH3470	This study
DRH3731	C6706 *rpsL*^{Sm} *lacZ flrC*::ΔTn/pDRH3471	This study
DRH3753	C6706 *rpsL*^{Sm} *lacZ fliF*::ΔTn/pDRH3470	This study
DRH3756	C6706 *rpsL*^{Sm} *lacZ fliF*::ΔTn/pDRH3471	This study
DRH3758	C6706 *rpsL*^{Sm} *lacZ fliF*::ΔTn/pDRH3472	This study
DRH3766	C6706 *rpsL*^{Sm} *lacZ flhB*::ΔTn/pDRH3470	This study
DRH3768	C6706 *rpsL*^{Sm} *lacZ flhB*::ΔTn/pDRH3471	This study
DRH3770	C6706 *rpsL*^{Sm} *lacZ flhB*::ΔTn/pDRH3472	This study
DRH3777	C6706 *rpsL*^{Sm} *lacZ fliP*::ΔTn/pDRH3470	This study
DRH3780	C6706 *rpsL*^{Sm} *lacZ fliP*::ΔTn/pDRH3471	This study
DRH3801	C6706 *rpsL*^{Sm} *lacZ fliP*::ΔTn/pDRH3472	This study
DRH3808	C6706 *rpsL*^{Sm} *lacZ fliR*::ΔTn/pDRH3470	This study
DRH3812	C6706 *rpsL*^{Sm} *lacZ fliR*::ΔTn/pDRH3471	This study
DRH3815	C6706 *rpsL*^{Sm} *lacZ fliR*::ΔTn/pDRH3472	This study
DRH3905	C6706 *rpsL*^{Sm} *lacZ* Δ*fliQ*	This study
DRH3918	C6706 *rpsL*^{Sm} *lacZ*/pDRH3474	This study
DRH3921	C6706 *rpsL*^{Sm} *lacZ rpoN*::ΔTn/pDRH3474	This study
DRH3924	C6706 *rpsL*^{Sm} *lacZ fliA*::ΔTn/pDRH3474	This study
DRH3926	C6706 *rpsL*^{Sm} *lacZ flrA*::ΔTn/pDRH3474	This study
DRH3932	C6706 *rpsL*^{Sm} *lacZ flrC*::ΔTn/pDRH3474	This study
DRH3936	C6706 *rpsL*^{Sm} *lacZ flhB*::ΔTn/pDRH3474	This study
DRH3940	C6706 *rpsL*^{Sm} *lacZ fliP*::ΔTn/pDRH3474	This study
DRH3945	C6706 *rpsL*^{Sm} *lacZ fliF*::ΔTn/pDRH3474	This study
DRH3953	C6706 *rpsL*^{Sm} *lacZ* Δ*fliG*	This study

DRH3958	C6706 *rpsL*^{Sm} *lacZ* Δ*fliN*	This study
DRH3967	C6706 *rpsL*^{Sm} *lacZ* Δ*fliG*/pDRH3470	This study
DRH3972	C6706 *rpsL*^{Sm} *lacZ* Δ*fliG*/pDRH3471	This study
DRH3974	C6706 *rpsL*^{Sm} *lacZ* Δ*fliG*/pDRH3472	This study
DRH3979	C6706 *rpsL*^{Sm} *lacZ* Δ*fliG*/pDRH3474	This study
DRH4001	C6706 *rpsL*^{Sm} *lacZ* Δ*fliN*/pDRH3470	This study
DRH4004	C6706 *rpsL*^{Sm} *lacZ* Δ*fliN*/pDRH3471	This study
DRH4007	C6706 *rpsL*^{Sm} *lacZ* Δ*fliN*/pDRH3472	This study
DRH4014	C6706 *rpsL*^{Sm} *lacZ* Δ*fliQ*/pDRH3471	This study
DRH4018	C6706 *rpsL*^{Sm} *lacZ* Δ*fliQ* /pDRH3470	This study
DRH4024	C6706 *rpsL*^{Sm} *lacZ* Δ*fliQ*/pDRH3472	This study
DRH4028	C6706 *rpsL*^{Sm} *lacZ* Δ*fliQ*/pDRH3474	This study
DRH4043	C6706 *rpsL*^{Sm} *lacZ fliR*::ΔTn/pDRH3474	This study
DRH4076	C6706 *rpsL*^{Sm} *lacZ* Δ*flhA*	This study
DRH4081	C6706 *rpsL*^{Sm} *lacZ* Δ*fliM*	This study
DRH4101	C6706 *rpsL*^{Sm} *lacZ* Δ*flhA*/pDRH3470	This study
DRH4103	C6706 *rpsL*^{Sm} *lacZ* Δ*fliN*/pDRH3474	This study
DRH4105	C6706 *rpsL*^{Sm} *lacZ* Δ*flhA*/pDRH3471	This study
DRH4107	C6706 *rpsL*^{Sm} *lacZ* Δ*flhA*/pDRH3472	This study
DRH4113	C6706 *rpsL*^{Sm} *lacZ* Δ*flhA*/pDRH3474	This study
DRH4117	C6706 *rpsL*^{Sm} *lacZ* Δ*fliM*/pDRH3470	This study
DRH4121	C6706 *rpsL*^{Sm} *lacZ* Δ*fliM*/pDRH3471	This study
DRH4123	C6706 *rpsL*^{Sm} *lacZ* Δ*fliM*/pDRH3472	This study
DRH4128	C6706 *rpsL*^{Sm} *lacZ* Δ*fliM*/pDRH3474	This study
DRH5560	C6706 *rpsL*^{Sm} *lacZ* Δ*flhG*	This study
DRH5570	C6706 *rpsL*^{Sm} *lacZ fliEp-flgKL*	This study
DRH5574	C6706 *rpsL*^{Sm} *lacZ* Δ*flhG fliEp-flgKL*	This study
DRH6903	C6706 *rpsL*^{Sm} *lacZ fliEp -flgBCDE*	This study
DRH6909	C6706 *rpsL*^{Sm} *lacZ* Δ*flhG fliEp-flgBCDE*	This study
DRH6910	C6706 *rpsL*^{Sm} *lacZ fliEp-flgBCDE fliEp-flgKL*	This study
DRH6915	C6706 *rpsL*^{Sm} *lacZ* Δ*flhG fliEp-flgBCDE fliEp-flgKL*	This study
DRH6926	C6706 *rpsL*^{Sm} *lacZ fliEp-flgFGHIJ*	This study
DRH6928	C6706 *rpsL*^{Sm} *lacZ* Δ*flhG fliEp-flgFGHIJ*	This study
DRH6931	C6706 *rpsL*^{Sm} *lacZ fliEp-flgBCDE fliEp-flgFGHIJ*	This study
DRH6935	C6706 *rpsL*^{Sm} *lacZ* Δ*flhG fliEp-flgBCDE fliEp-flgFGHIJ*	This study
DRH6939	C6706 *rpsL*^{Sm} *lacZ fliEp-flgFGHIJ fliEp flgKL*	This study
DRH6942	C6706 *rpsL*^{Sm} *lacZ* Δ*flhG fliEp-flgFGHIJ fliEp-flgKL*	This study
DRH6945	C6706 *rpsL*^{Sm} *lacZ fliEp-flgBCDE fliEp-flgFGHIJ fliEp-flgKL*	This study
DRH6948	C6706 *rpsL*^{Sm} *lacZ* Δ*flhG fliEp-flgBCDE fliEp-flgFGHIJ fliEp-flgKL*	This study
PMB363	C6706 *rpsL*^{Sm} *lacZ fliG* $_{\Delta NTD}$	This study

PMB364	C6706 *rpsL*^{Sm} *lacZ fliG* $_{\Delta MD}$	This study
PMB370	C6706 *rpsL*^{Sm} *lacZ fliG* $_{\Delta CTD}$	This study
PMB375	C6706 *rpsL*^{Sm} *lacZ fliG* $_{\Delta NTD}$/pDRH3470	This study
PMB378	C6706 *rpsL*^{Sm} *lacZ fliG* $_{\Delta MD}$/pDRH3470	This study
PMB404	C6706 *rpsL*^{Sm} *lacZ fliG* $_{\Delta CTD}$/pDRH3470	This study
PMB1079	C6706 *rpsL*^{Sm} *lacZ rpoN*::ΔTn/pDRH6921	This study
PMB1101	C6706 *rpsL*^{Sm} *lacZ fliA*::ΔTn/pDRH6921	This study
PMB1105	C6706 *rpsL*^{Sm} *lacZ flrA*::ΔTn/pDRH6921	This study
PMB1110	C6706 *rpsL*^{Sm} *lacZ flrB*::ΔTn/pDRH6921	This study
PMB1114	C6706 *rpsL*^{Sm} *lacZ flrC*::ΔTn/pDRH6921	This study
PMB1152	C6706 *rpsL*^{Sm} *lacZ* /pDRH6921	This study
PMB1157	C6706 *rpsL*^{Sm} *lacZ* /pDRH6924	This study
PMB1166	C6706 *rpsL*^{Sm} *lacZ* Δ*flhG*/pDRH6924	This study
PMB1210	C6706 *rpsL*^{Sm} *lacZ* /pDRH6925	This study
PMB1218	C6706 *rpsL*^{Sm} *lacZ* Δ*flhG*/pDRH6925	This study
PMB1244	C6706 *rpsL*^{Sm} *lacZ* Δ*flhG*/pDRH6921	This study
PMB1265	C6706 *rpsL*^{Sm} *lacZ* Δ*flhA*/pDRH6921	This study
PMB1269	C6706 *rpsL*^{Sm} *lacZ flhB*::ΔTn/pDRH6921	This study
PMB1272	C6706 *rpsL*^{Sm} *lacZ fliP*::ΔTn/pDRH6921	This study
PMB1276	C6706 *rpsL*^{Sm} *lacZ* Δ*fliQ*/pDRH6921	This study
PMB1280	C6706 *rpsL*^{Sm} *lacZ fliR*::ΔTn/pDRH6921	This study
PMB1303	C6706 *rpsL*^{Sm} *lacZ fliF*::ΔTn/pDRH6921	This study
PMB1309	C6706 *rpsL*^{Sm} *lacZ* Δ*fliG*/pDRH6921	This study
PMB1313	C6706 *rpsL*^{Sm} *lacZ* Δ*fliM*/pDRH6921	This study
PMB1316	C6706 *rpsL*^{Sm} *lacZ* Δ*fliN*/pDRH6921	This study
PMB1535	C6706 *rpsL*^{Sm} *lacZ fliF*::ΔTn/pDRH3470 + pDRH6973	This study
PMB1579	C6706 *rpsL*^{Sm} *lacZ fliF*::ΔTn/pDRH6972	This study
PMB1581	C6706 *rpsL*^{Sm} *lacZ fliF*::ΔTn/pDRH6973	This study
PMB1611	C6706 *rpsL*^{Sm} *lacZ fliF*::ΔTn/pDRH3470 + pDRH6972	This study
PMB1812	C6706 *rpsL*^{Sm} *lacZ* Δ*flhG*/pDRH3470	This study
PMB1816	C6706 *rpsL*^{Sm} *lacZ* Δ*flhG*/pDRH3470	This study
PMB1817	C6706 *rpsL*^{Sm} *lacZ* Δ*flhG*/pDRH3474	This study
PMB1822	C6706 *rpsL*^{Sm} *lacZ* Δ*flhG*/pDRH3471	This study
WPK134	C6706 *rpsL*^{Sm} *lacZ*/pDRH3472	This study
WPK136	C6706 *rpsL*^{Sm} *lacZ flrA*::ΔTn/pDRH3470 + pDRH4912	This study
WPK139	C6706 *rpsL*^{Sm} *lacZ fliR*::ΔTn/pDRH3470 + pDRH4910	This study
WPK142	C6706 *rpsL*^{Sm} *lacZ fliR*::ΔTn/pDRH3470 + pDRH4916	This study
WPK146	C6706 *rpsL*^{Sm} *lacZ* Δ*fliQ*/pDRH3470 + pDRH4910	This study
WPK149	C6706 *rpsL*^{Sm} *lacZ* Δ*fliQ*/pDRH3470 + pDRH4915	This study
WPK153	C6706 *rpsL*^{Sm} *lacZ flrB*::ΔTn/pDRH3470 + pDRH4910	This study
WPK156	C6706 *rpsL*^{Sm} *lacZ flrA*::ΔTn/pDRH3470 + pDRH4910	This study

WPK161	C6706 *rpsL*^{Sm} *lacZ flrB*::ΔTn/pDRH3470 + pDRH4913	This study
WPK170	C6706 Δ*flhA*/pDRH3470 + pWPK165	This study
WPK173	C6706 *rpsL*^{Sm} *lacZ flhB*::ΔTn/pDRH3470 + pWPK166	This study
WPK175	C6706 *rpsL*^{Sm} *lacZ flrC*::ΔTn/pDRH3470 + pWPK163	This study
WPK178	C6706 *rpsL*^{Sm} *lacZ* Δ*fliG*/pDRH3470 + pWPK164	This study
WPK204	C6706 *rpsL*^{Sm} *lacZ fliF*::ΔTn/pDRH3470 + pWPK168	This study
WPK208	C6706 *rpsL*^{Sm} *lacZ* Δ*flhA*/pDRH3470 + pDRH4910	This study
WPK209	C6706 *rpsL*^{Sm} *lacZ flhB*::ΔTn/pDRH3470 + pDRH4910	This study
WPK214	C6706 *rpsL*^{Sm} *lacZ flrC*::ΔTn/pDRH3470 + pDRH4910	This study
WPK216	C6706 *rpsL*^{Sm} *lacZ* Δ*fliG*/pDRH3470 + pDRH4910	This study
WPK221	C6706 *rpsL*^{Sm} *lacZ fliF*::ΔTn/pDRH3470 + pDRH4910	This study
***Pseudomonas aeruginosa* strains**		
PA14	Wild-type; UCBPP-PA14	(13, 14)
SMC1253	PA14 *rpoN*::Gm; Wild-type; UCBPP-PA14	(15)
DRH4335	PA14 *att*::*flgB*$_{p}$*-lacZ*	This study
DRH4413	PA14 Δ*flhB att*::*flgBp-lacZ*	This study
DRH4420	PA14 Δ*fliP att*::*flgBp-lacZ*	This study
DRH4440	PA14 Δ*fliG att*::*flgBp-lacZ*	This study
DRH4443	PA14 *att*::*fliAp-lacZ*	This study
DRH4457	PA14 Δ*fleS att*::*fliA*$_{p}$*-lacZ*	This study
DRH4607	PA14 Δ*fleS att*::*flgBp-lacZ*	This study
DRH4613	PA14 Δ*fleQ att*::*flgBp-lacZ*	This study
DRH4716	PA14 Δ*fleQ att*::*flgBp-lacZ*	This study
DRH4620	PA14 Δ*fliF att*::*flgBp-lacZ*	This study
DRH4625	PA14 Δ*fliA att*::*flgBp-lacZ*	This study
DRH4633	PA14 Δ*flhA att*::*flgBp-lacZ*	This study
DRH4637	PA14 Δ*fliA*	This study
DRH4641	PA14 Δ*fleS*	This study
DRH4644	PA14 Δ*fleQ*	This study
DRH4648	PA14 Δ*fleR*	This study
DRH4651	PA14 Δ*fliM*	This study
DRH4654	PA14 Δ*fliG*	This study
DRH4657	PA14 Δ*flhA*	This study
DRH4660	PA14 Δ*flhB*	This study
DRH4663	PA14 Δ*fliO*	This study
DRH4666	PA14 Δ*fliR*	This study
DRH4669	PA14 Δ*fliQ*	This study
DRH4672	PA14 Δ*fliP*	This study
DRH4675	PA14 Δ*fliF*	This study
DRH4678	PA14 Δ*fliN*	This study
DRH4707	PA14 Δ*fliA att*::*fliAp-lacZ*	This study

DRH4725	PA14 Δ*fleR att*::*fliAp-lacZ*	This study
DRH4734	PA14 Δ*fliM att*::*fliAp-lacZ*	This study
DRH4740	PA14 Δ*fliG att*::*fliAp-lacZ*	This study
DRH4750	PA14 Δ*fliM att*::*flgBp-lacZ*	This study
DRH4772	PA14 Δ*fliO att*::*flgBp-lacZ*	This study
DRH4824	PA14 Δ*flhA att*::*flgBp-lacZ*	This study
DRH4826	PA14 Δ*flhB att*::*fliAp-lacZ*	This study
DRH4829	PA14 Δ*fliR att*::*fliAp-lacZ*	This study
DRH4835	PA14 Δ*fliQ att*::*flgBp-lacZ*	This study
DRH4837	PA14 Δ*fliR att*::*flgBp-lacZ*	This study
DRH4841	PA14 Δ*fliN att*::*flgBp-lacZ*	This study
DRH4867	PA14 Δ*fliO att*::*fliAp-lacZ*	This study
DRH4870	PA14 Δ*fliQ att*::*fliAp-lacZ*	This study
DRH4874	PA14 Δ*fliP att*::*fliAp-lacZ*	This study
DRH4876	PA14 Δ*fliF att*::*fliAp-lacZ*	This study
DRH4880	PA14 Δ*fliN att*::*fliAp-lacZ*	This study
DRH5063	PA14 *fliG* ΔNTD *att*::*flgBp-lacZ*	This study
DRH5044	PA14 *fliG* ΔMD *att*::*flgBp-lacZ*	This study
DRH5048	PA14 *fliG* ΔCTD *att*::*flgBp-lacZ*	This study
DRH5818	PA14 *rpoN::*Gm *att*::*flgBp-lacZ*	This study
DRH5820	PA14 *rpoN*::Gm *att*::*fliAp-lacZ*	This study
