# Supplementary material for: A Polar Flagellar Transcriptional Program Mediated by Diverse Two-Component Signal Transduction Systems and Basal Flagellar Proteins Is Broadly Conserved in Polar Flagellates
Source: mBio. 2020 Mar 3;11(2):e03107-19. doi: 10.1128/mBio.03107-19 (PMC7064773; doi:10.1128/mBio.03107-19)
Supplement: TABLE S2 [file mBio.03107-19-st002.pdf]

Table S2. Plasmids used in this study

Strain	Genotype	Source/Reference
**Plasmids for recombinant protein production**		
pGEX4T-2	Amp^R; GST-fusion vector	GE Healthcare
pQE30	Amp^R; for construction of N-terminal 6XHis tag fusions	Qiagen
**Plasmids for construction of *Campylobacter jejuni* strains**		
pDRH424	pUC19 containing *astA*::*cat-rpsL*	(2)
pDRH449	pUC19 containing Δ*astA*	(2)
pDRH665	pUC19 containing *flaB*::*astA*	(2)
**Plasmids for construction of *Vibrio cholerae* strains**		
pTL61T	Amp^R; for creation of *lacZ* transcriptional reporters to be expressed *in trans* in *V. cholerae*	(16)
pFlpE	Amp^R; encodes Flp recombinase	(3)
pACYC184	Amp^R; Tc^R; complementation vector for *V. cholerae*	(17, 18)
pGP704sacB28	Amp^R; *sacB*$^+$; *mob/oriT*; suicide vector for creation of *V. cholerae* mutants	G. Schoolnik
pKAS32	Amp^R; *rpsL*$^+$; *mob/oriT*; suicide vector for creation of *V. cholerae* mutants	(19)
pDRH3470	pTL61T with *flaA* promoter region from nucleotides 6-906 upstream of the *flaA* start codon cloned into the SalI and BamHI sites to create *flaAp-lacZ* transcriptional fusion	This study
pDRH3472	pTL61T with *cheV* promoter region from nucleotides 7-714 upstream of the *cheV* start codon cloned into the SalI and BamHI sites to create *cheVp-lacZ* transcriptional fusion	This study
pDRH3474	pTL61T with *flgB* promoter region from nucleotides 7-726 upstream of the *flgB* start codon cloned into the SalI and BamHI sites to create *flgBp-lacZ* transcriptional fusion	This study
pDRH3476	pKAS32 with DNA fragment to create in-frame deletion of codons 7-342 of *fliG* with 0.75 kb of upstream and downstream sequence cloned into the XbaI site	This study
pDRH3477	pKAS32 with DNA fragment to create in-frame deletion of codon 2-109 of *fliN* with 0.75 kb of upstream and downstream sequence cloned as a BamHI fragment into the BglII site	This study
pDRH3478	pKAS32 with DNA fragment to create in-frame deletion of codons 2-81 of *fliG* with 0.75 kb of upstream and downstream sequence cloned as a BamHI fragment into the BglII site	This study

pDRH4064	pKAS32 with DNA to create in-frame deletion of codons 2-681 of *flhA* with 0.75 kb of upstream and downstream sequence cloned into the XbaI site	This study
pDRH4069	pKAS32 with DNA to create in-frame deletion of codons 20-330 of *fliM* with 0.75 kb of upstream and downstream sequence cloned into the XbaI site	This study
pDRH4505	pGEX4T-2 with *flrB* from codon 2 to stop codon cloned into the BamHI and SalI sites of pGEX4T-2	This study
pDRH4532	pQE30 with *fliF* from codons 45 to 473 encoding the periplasmic region cloned ito the BamHI and SalI sites	This study
pDRH4910	pACYC184 digested with EcoRV and NruI and re-ligated to delete a portion of the tetracycline resistance gene	This study
pDRH4912	pACYC184 with *flrA* RBS and coding sequence cloned into the BamHI and SalI sites	This study
pDRH4913	pACYC184 with *flrB* RBS and coding sequence cloned into the BamHI and SalI sites	This study
pDRH4915	pACYC184 with *fliQ* RBS and coding sequence cloned into the BamHI and SalI sites	This study
pDRH4916	pACYC184 with *fliR* RBS and coding sequence cloned into the BamHI and EcoRV sites	This study
pDRH4952	pGEX4T-2 with *fliG* from codon 2 to stop codon cloned into the SmaI site	This study
pDRH5507	pGP704sacB28 with DNA fragment to create in-frame deletion of codons 265-248 of *fliG* with 0.75 kb of upstream and downstream sequence cloned into the XbaI site	This study
pDRH5512	pGP704sacB28 with DNA fragment to create in-frame deletion of codons 166-263 of *fliG* with 0.75 kb of upstream and downstream sequence cloned into the XbaI site	This study
pDRH5523	pGP704sacB28 with DNA fragment to create in-frame deletion of codons 6-165 of *fliG* with 0.75 kb of upstream and downstream sequence cloned into the XbaI site	This study
pDRH5545	pGEX-4T-2 with *rpoA* cloned from codon 2 to stop codon cloned into the BamHI	This study
pDRH5564	pGP704sacB28 with DNA fragment that fuses the nucleotides -817 to -162 upstream of the *flgK* start codon to nucleotides -89 to -1 of *fliE* containing the *fliEp* to the coding sequence of *flgK* cloned into the XbaI site	This study
pDRH5578	pGP704sacB28 with DNA fragment that fuses the nucleotides -998 to -195 upstream of the *flgB* start codon to nucleotides -89 to -1 of *fliE* containing the	This study

	fliEp to the coding sequence of *flgB* cloned into the XbaI site	
pDRH5564	pGP704sacB28 with DNA fragment that fuses the nucleotides -1005 to -158 upstream of the *flgF* start codon to nucleotides -89 to -1 of *fliE* containing the *fliEp* to the coding sequence of *flgF* cloned into the XbaI site	This study
pDRH6921	pTL61T with *flgF* promoter region from nucleotides 7-741 upstream of the *flgF* start codon cloned into the SalI and BamHI sites to create *flgFp*-*lacZ* transcriptional fusion	This study
pDRH6923	pTL61T with *fliE* promoter region from nucleotides 7-742 upstream of the *fliE* start codon cloned into the SalI and BamHI sites to create *fliEp*-*lacZ* transcriptional fusion	This study
pDRH6924	pTL61T with *flrA* promoter region from nucleotides 8-624 upstream of the *flrA* start codon cloned into the SalI and XbaI sites to create *flrAp*-*lacZ* transcriptional fusion	This study
pDRH6925	pTL61T with *flrB* promoter region from nucleotides 9-781 upstream of the *flrB* start codon cloned into the SalI and BamHI sites to create *flrBp*-*lacZ* transcriptional fusion	This study
pDRH6972	pACYC184 with $fliF_{\Delta AS200\text{-}201}$ RBS and coding sequence cloned into the BamHI and SalI sites	This study
pDRH6973	pACYC184 with $fliF_{\Delta AS202\text{-}203}$ RBS and coding sequence cloned into the BamHI and SalI sites	This study
pWPK163	pACYC184 with *flrC* RBS and coding sequence cloned into the BamHI and SalI sites	This study
pWPK164	pACYC184 with *fliG* RBS and coding sequence cloned into the EcoRV site	This study
pWPK165	pACYC184 with *flhA* RBS and coding sequence cloned into the BamHI and SalI sites	This study
pWPK166	pACYC184 with *flhB* RBS and coding sequence cloned into the BamHI and SalI sites	This study
pWPK168	pACYC184 with *fliF* RBS and coding sequence cloned into the BamHI and SalI sites	This study
**Plasmids for construction of *Pseudomonas aeruginosa* strains**		
mini-CTX-*lacZ*	Tc^R; single copy integration vector at *att* site containing a promoterless *lacZ*; Tc	(20)
pEX18Gm	Gm^R; *oriT*$^+$ *sacB*$^+$; suicide vector for creation of *Pseudomonas aeruginosa* mutants	(21)
pFLP2	Amp^R; contains gene for Flp for recombination at FRT sites to remove mini-CTX backbone from chromosome	(21)

pDRH4315	mini-CTX-*lacZ* with *flgB* promoter region from nucleotides 7-721 upstream of the *flgB* start codon cloned into the SalI and BamHI sites to create *flgBp-lacZ* transcriptional fusion	This study
pDRH4341	pEX18Gm with DNA fragment to create in-frame deletion of codons 4-236 of *fliP* with 0.75 kb of upstream and downstream sequence cloned into the XbaI site	This study
pDRH4343	pEX18Gm with DNA fragment to fuse the start and stop codons of *flhB* with 0.75 kb of upstream and downstream sequence cloned into the XbaI site	This study
pDRH4348	pEX18Gm with DNA fragment to delete the entire coding sequence of *fleQ* with 0.75 kb of upstream and downstream sequence cloned into the XbaI site	This study
pDRH4352	pEX18Gm with DNA fragment to create in-frame deletion of codons 2-368 of *fleS* with 0.75 kb of upstream and downstream sequence cloned into the XbaI site	This study
pDRH4356	pEX18Gm with DNA fragment to create in-frame deletion of codons 2-305 of *fliG* with 0.75 kb of upstream and downstream sequence cloned into the XbaI site	This study
pDRH4365	pEX18Gm with DNA fragment to delete the entire coding sequence of *fleR* with 0.75 kb of upstream and downstream sequence cloned into the XbaI site	This study
pDRH4366	pEX18Gm with DNA fragment to create in-frame deletion of codons 2-215 of *fliA* with 0.75 kb of upstream and downstream sequence cloned into the XbaI site	This study
pDRH4371	pEX18Gm with DNA fragment to create in-frame deletion of codons 2-699 of *flhA* with 0.75 kb of upstream and downstream sequence cloned into the XbaI site	This study
pDRH4374	pEX18Gm with DNA fragment to create in-frame deletion of codons 3-225 of *fliR* with 0.75 kb of upstream and downstream sequence cloned into the XbaI site	This study
pDRH4376	pEX18Gm with DNA fragment to create in-frame deletion of codons 2-116 of *fliO* with 0.75 kb of upstream and downstream sequence cloned into the XbaI site	This study
pDRH4377	pEX18Gm with DNA fragment to create in-frame deletion of codons 2-564 of *fliF* with 0.75 kb of upstream and downstream sequence cloned into the XbaI site	This study

pDRH4379	pEX18Gm with DNA fragment to create in-frame deletion of codons 2-289 of *fliM* with 0.75 kb of upstream and downstream sequence cloned into the XbaI site	This study
pDRH4381	pEX18Gm with DNA fragment to create in-frame deletion of codons 2-124 of *fliN* with 0.75 kb of upstream and downstream sequence cloned into the XbaI site	This study
pDRH4470	mini-CTX-*lacZ* with *fliA* promoter region from nucleotides 7-796 upstream of the *flgB* start codon cloned into the SalI and BamHI sites to create *flgBp-lacZ* transcriptional fusion	This study
pDRH4474	pEX18Gm with DNA fragment to create in-frame deletion of codons 2-56 of *fliQ* with 0.75 kb of upstream and downstream sequence cloned into the XbaI site	This study
pDRH4526	pQE30 with *fliG* coding sequence from codon 2 to stop codon cloned into the BamHI and SmaI sites of pQE30	This study
pDRH5033	pEX18Gm with DNA fragment to create in-frame deletion of codons 3-105 of *fliG* with 0.75 kb of upstream and downstream sequence cloned into the XbaI site	This study
pDRH5034	pEX18Gm with DNA fragment to create in-frame deletion of codons 106-202 of *fliG* with 0.75 kb of upstream and downstream sequence cloned into the XbaI site	This study
pDRH5035	pEX18Gm with DNA fragment to create in-frame deletion of codons 203-329 of *fliG* with 0.75 kb of upstream and downstream sequence cloned into the XbaI site	This study
